# Traditional Calibration Methods in Atomic Spectrometry and New Calibration Strategies for Inductively Coupled Plasma Mass Spectrometry

**DOI:** 10.3389/fchem.2018.00504

**Published:** 2018-11-13

**Authors:** Jake A. Carter, Ariane I. Barros, Joaquim A. Nóbrega, George L. Donati

**Affiliations:** ^1^Department of Chemistry, Wake Forest University, Winston-Salem, NC, United States; ^2^Group for Applied Instrumental Analysis, Department of Chemistry, Federal University of São Carlos, São Carlos, Brazil

**Keywords:** matrix-matched calibration, internal standardization, standard additions, interference standard method, standard dilution analysis, multi-isotope calibration, multispecies calibration

## Abstract

Applications, advantages, and limitations of the traditional external standard calibration, matrix-matched calibration, internal standardization, and standard additions, as well as the non-traditional interference standard method, standard dilution analysis, multi-isotope calibration, and multispecies calibration methods are discussed.

## Introduction

Instrumental spectrochemical methods are widely used in trace element analysis of all types of samples, and different calibration strategies such as external standard calibration (EC), matrix-matched calibration (MMC), internal standardization (IS), and standard additions (SA) are employed to overcome matrix effects and improve accuracy and precision. These traditional calibration methods are closely associated with several of the most commonly used spectroanalytical methods, as discussed here.

It is well-known that inductively coupled plasma mass spectrometry (ICP-MS) is severely affected by different interfering effects, and several strategies have been developed for overcoming them. In inductively coupled plasma optical emission spectrometry (ICP OES), non-spectral interferences can be related to transport effects during sample introduction, and plasma effects such as those caused by high concentrations of either carbon or easily-ionized elements. Interface processes, such as self-absorption in axial-viewing ICP OES, space charge effects in ICP-MS, and recombination processes in both ICP OES and ICP-MS, are also responsible for biased results. Spectral interferences are mainly related to processes involving nearby emission lines and background signals (ICP OES), isotopes with nearby masses (for the typical low-resolution quadrupole-based ICP-MS), double charge species (ICP-MS), and effects caused by polyatomic ions (ICP OES and ICP-MS). In quadrupole-based ICP-MS (ICP-QMS), these effects can be corrected by using specially designed instrumentation containing a collision/reaction cell (CRC) with a single quadrupole, or a tandem arrangement with two quadrupoles and a CRC. A simpler and cost-effective alternative to correct for interfering effects in both ICP OES and ICP-MS is the use of special strategies for calibration.

In this review paper, we discuss applications, advantages and limitations of traditional and non-traditional calibration methods. The discussions associated with the traditional calibration strategies involve several instrumental spectrochemical methods. On the other hand, the non-traditional calibration strategies examined here, which include the interference standard method (IFS), standard dilution analysis (SDA), multi-isotope calibration (MICal), and multispecies calibration (MSC), are focused on ICP-MS applications. All discussions are based on landmark papers and recent works describing the state of the art of new approaches. A careful reading of this literature suggests that several interfering effects can be controlled, without any instrument modification, by fully exploiting data acquisition and processing. Thus, simple calibration strategies may be able to improve accuracy and precision in trace element analysis.

## Traditional calibration methods

### The external standard (EC) and matrix-matched calibration (MMC) methods

In analytical chemistry, calibration involves the determination of a mathematical function describing the relationship between analyte concentration and instrument response. A series of calibration standards is used to determine this function, which is then applied to determine the unknown amount of analyte in a sample (Currie, 1998; Cuadros-Rodríguez et al., 2001; AOAC International, 2002). Usually, calibration is performed in three steps: standard solution preparation, analytical signal measurement, and mathematical calculation (i.e., determination of the calibration function model; Kościelniak, 2003). The first step will depend on the type of calibration method used. The traditional EC, MMC, IS, and SA methods are the most commonly used calibration strategies, with broad application in quantitative analytical chemistry.

The EC method is the most straightforward, and therefore, the most used among the traditional calibration strategies. It is called external standard calibration because certified pure substances or standard solutions used in the calibration procedure are external to the sample. It assumes matrix effects are absent or have negligible impact on the analytical signal. In many applications, this is in fact the case, and EC can be successfully applied by simply comparing the analytical signals from standard solutions with those from samples. In principle, EC can be performed using single, double or multiple calibration standards (Cuadros-Rodríguez et al., 2001). However, better precision and accuracy are achieved by adopting a multiple-point calibration (Miller and Miller, 1984). In this case, a set of standard solutions are analyzed, and analyte concentration vs. instrument response are plotted as independent variable (*x*-axis) and dependent variable (*y*-axis), respectively. The analyte concentration in the sample can then be determined by interpolation using the mathematical function obtained from the calibration plot, which is usually determined using least-squares regression. The instrument response recorded for the sample is converted into analyte concentration using a relationship such as *IR* = *a* + *bC*, where *IR, a, b*, and *C* correspond to instrument response, *y*-intercept, slope, and analyte concentration, respectively (Cuadros-Rodríguez et al., 2001). It is recommended by the AOAC International (2002) that the analyte concentrations in the standard solutions should be close to the one in the sample, and that 6–8 standard concentrations should be used for calibration.

In EC, as well as in other strategies, the choice of the calibration model depends on the characteristics of the data obtained (Currie, 1998). Linearity (or linear dynamic range), for example, can be tested graphically by a residual plot, or numerically by a correlation coefficient (*r*). One can also apply different statistical tools to check for linearity such as, for instance, the *F*-test. When employing this statistical method, the ratio between residual standard deviations of the linear model and those of a non-linear model is determined. If *F* calculated (*F*_*cal*_) is lower than *F* tabulated (*F*_*tab*_, usually at a 95% confidence level), the model is considered linear. On the other hand, if *F*_*cal*_ > *F*_*tab*_, a non-linear model (e.g., a quadratic model) should be used (Currie, 1998; Mermet, 2010; Raposo, 2016).

Ordinary least-squares (OLS) is a linear model based on minimization of the sum of squares of deviations (i.e., the difference between expected values and experimental data; Currie, 1998; Mermet, 2010). It can only be applied when variables are normally distributed, the error in concentration values (independent variable) is minimal when compared with the analytical signal error (dependent variable), and when the data are homoscedastic (Mermet, 2010). Homoscedasticity occurs when a sequence of random data presents homogeneous variance. To evaluate whether variations are homogeneous, statistical methods such as the Hartley and Bartlett test (Currie, 1998) are commonly employed. In most cases, data obtained from consolidated spectroanalytical instrumental methods are normally distributed and homoscedastic, and errors in standard solution concentrations are negligible, hence the OLS approach can be successfully applied in conjunction with EC.

Heteroscedasticity occurs when variances are heterogeneous, in which case OLS cannot be applied, and alternative models such as the weighted least-squares (WLS) must be used (Currie, 1998; Mermet, 2010). Different from OLS, in which all calibration points have equal weights, lower-concentration standards have the highest weights in WLS, which results in increased accuracy for those calibration points. As discussed by Taylor and Schutyser (1986), the analyst needs to be thoughtful and know which calibration model the software of an instrument is using to avoid inaccurate results.

In elemental analyses by ICP OES, ICP-MS, flame atomic absorption spectrometry (F AAS), and graphite furnace atomic absorption spectrometry (GF AAS), it is frequently necessary to prepare the sample in a manner that makes it compatible with the sample introduction system. Ideally, samples are converted into a solution, with minimal or no matrix present, so EC can be successfully used. The EC method has been effective in several analytical applications involving elemental determination in different types of the matrices, such as crude oils (Trevelin et al., 2016; Sugiyama and Williams-Jones, 2018), diesel oil (Nora et al., 2017), biodiesel (Barros et al., 2017), iron supplements (Barbosa et al., 2015), vinegar alcohol (Silva Junior et al., 2015), sediment (Leao et al., 2016b), milk (Oliveira et al., 2017), canned foods (Leao et al., 2016a), seawater, mineral water, tap water, cachaça, human blood (Papai et al., 2017), among others. In these applications, acid digestion (Barbosa et al., 2015; Leao et al., 2016a; Sugiyama and Williams-Jones, 2018), dry decomposition (Nora et al., 2017), extraction procedures (Silva Junior et al., 2015; Leao et al., 2016b; Sánchez et al., 2016; Trevelin et al., 2016; Barros et al., 2017; Papai et al., 2017; Zeiner et al., 2018), and emulsion formation (Oliveira et al., 2017) were used for sample preparation. Table 1 shows a summary and additional details on sample preparation procedures used in combination with EC, as well as with MMC.

**Table 1:** Sample preparation procedures and direct sample analysis used in combination with EC or MMC in elemental determinations.

**Sample**	**Sample preparation**	**Calibration method**	**Remarks**	**References**
Crude oil	Ashing and chemical oxidation/acid digestion	EC	Ashing (heating in muffle) and oxidation with nitric, hydrochloric acid, and hydrogen peroxide were carried out using the single-vessel strategy	Sugiyama and Williams-Jones, 2018
	Extraction induced by emulsion	EC	Extraction was induced by emulsion with xylene and Triton X-100 in HNO_3_, with posterior breaking of the emulsion by heat. The acid aqueous phase in the emulsion was analyzed, and calibration was performed using inorganic aqueous standards	Trevelin et al., 2016
Vegetable oils and biodiesel	Dilution with methyl isobutyl ketone 40% (v/v)	EC	The matrix effect was evaluated by comparing calibration solutions in organic and aqueous media. It was demonstrated that external standard calibration was possible	Almeida et al., 2016
Diesel oil	Microwave-induced combustion with diluted nitric acid (4 mol/L)	EC	Standards solutions used for calibration of ICP-MS were prepared in 0.7 mol/L HNO_3_	Nora et al., 2017
Biodiesel	Extraction with nitric acid (1% v/v) and manual agitation for 4 min	EC	Sample preparation allowed the use of inorganic aqueous standards for calibration rather than the organometallic standards recommended by the official method (ABNT NBR 15556, 2008)	Barros et al., 2017
Iron supplements	Digestion in heating block using nitric acid and hydrogen peroxide	EC	Direct analysis was not feasible because of soot formed after pyrolysis. Sugar in the samples produced soot, which caused radiation scattering and signal bias	Barbosa et al., 2015
Seawater, mineral water, tap water, cachaça, and human blood	Liquid phase extraction to solid phase extraction	EC	Copper(II) in aqueous samples was converted into a hydrophobic complex of copper(II) diethyldithiocarbamate and subsequently extracted into paraffin wax. The external standard calibration curve was obtained using the same extraction procedure with a standard Cu solution replacing the sample	Papai et al., 2017
Alcohol vinegar	Ultrasound-assisted extraction using thiourea as a chelating agent	EC	After sample preparation, hydrides were generated using isoamyl alcohol as an antifoaming agent and sodium tetrahydroborate. The vapor containing the hydrides was carried into a quartz T-tube, which was coupled to the AA spectrometer. The calibration standards were prepared in 0.05% (v/v) HNO_3_ and Hg was determined by cold vapor atomic absorption spectrometry (CV AAS)	Silva Junior et al., 2015
Sediment	–	EC	–	Leao et al., 2016b
Milk	Milk sample slurries using Triton X-100 and HNO_3_	EC	Results for EC and SA were similar, indicating that there was no matrix interference. EC was chosen for Pb determination by GFAAS due to its simplicity and lower cost, and to extend the lifetime of the graphite tube	Oliveira et al., 2017
Canned foods (sardine and tomato)	Microwave-assisted digestion with HNO_3_ and H_2_O_2_	EC	External standard calibration based on aqueous standard solutions	Leao et al., 2016a
Cheese	–	EC	Dry cheese samples were automatically weighed on a balance and introduced into the furnace. Pb concentrations were determined using aqueous standard solutions and HR-CS GF AAS	Tinas et al., 2017
Phosphate fertilizers	–	EC	External standard calibration with aqueous standard solutions (in 0.014 mol/L HNO_3_) was adopted for Cu and Hg determination by HR-CS-GF AAS	Souza et al., 2015
Spices	–	EC	The use of Pd/Mg(NO_3_)_2_ as chemical modifier for Cd, and an additional air-assisted pyrolysis step allowed the use of aqueous standard solutions for calibration of all analytes (Cd, Ni, and V)	Virgilio et al., 2015
Eye shadow	–	EC	The pyrolysis temperature was adequate to eliminate the complex matrix of the facial make-up samples, and external standard calibration using aqueous standard solutions was used to determine Cd, Pb, and Sb	Barros et al., 2014, 2015, 2016
Incense	–	EC and MMC	Matrix effects were evaluated by means of calibration standards prepared in the aqueous and solid media. EC with aqueous standard solutions, and MMC were selected for Pb determination in rods and coatings, respectively	Coco et al., 2017
High-purity Silicon	–	EC	Matrix effects were evaluated by comparing the slopes of calibration curves built up using aqueous standard solutions and solid media (SoG-Si). The similarities of the slopes indicated no matrix effects present, and EC was employed	Bechlin et al., 2016
Soil	–	EC and MMC	EC was effective for Mo, Ni, and V determination in soil samples using aqueous standard solutions. For Co determination, MMC with Montana Soil II CRM was required	Babos et al., 2017

In addition to the procedures mentioned before, there are other types of sample preparation which are also compatible with EC. For example, dilution of biodiesel with ethanol allows for using inorganic standards and aqueous solutions in elemental determination by microwave-induced plasma optical emission spectrometry (MIP OES) and EC (Amais et al., 2013). Dilution of biodiesel and vegetable oil with isobutyl ketone is also compatible with EC using aqueous standards and determination by high resolution-continuum source GF AAS (HR-CS GF AAS; Almeida et al., 2016). Photo-oxidation by UV radiation is another strategy to minimize matrix effects and allow for using inorganic-matrix aqueous standard solutions in fruit juice analysis by F AAS and EC (Brandão et al., 2012). When possible, simple dilution of aqueous samples makes the application of EC even more straightforward. Zhuravlev et al. (2016), for example, employed EC in elemental analysis of ground natural water with moderate salinity (6.79 g/L) after a 10-fold dilution with 5% (v/v) HNO_3_. For samples with high salinity (16.8, 34.8 g/L), 20-fold dilutions in 5% (v/v) HNO_3_ were employed, and analyte concentrations were determined by filter-furnace electrothermal atomic absorption spectroscopy (FF ET-AAS). For determinations using inductively coupled plasma sector field mass spectrometry (ICP-SFMS) and ICP-QMS, a 10-fold dilution was sufficient for all samples. For more complex matrices, an alternative to simple dilution or total digestion is the use extraction procedures. Veguería et al. (2013), for example, used EC and ICP-MS for seawater analysis after preconcentration and matrix elimination on iminodiacetate resins and elution with nitric acid.

Direct solid sampling and HR-CS GF AAS determination using aqueous standard solutions and EC may significantly improve sample throughput, as minimal sample preparation is required. As examples of such a strategy we have elemental analysis of cheese (Tinas et al., 2017), fertilizers (Souza et al., 2015), spices (Virgilio et al., 2015), eye shadow (Barros et al., 2014, 2015, 2016), incense (Coco et al., 2017), high-purity silicon (Bechlin et al., 2016), and soil (Babos et al., 2017; Table 1).

Although widely used due to its simplicity and flexibility, EC frequently provides inaccurate results when applied to complex-matrix sample analyses. In these cases, methods such as SA may be more appropriate to minimize signal bias and improve accuracy, as will be discussed in detail in the following sections. Li et al. (2016), for example, reported poor accuracy in honey analysis by electrothermal vaporization ICP-MS (ETV-ICP-MS), which was caused by sample transport differences between the honey homogenate and standard solutions, and by non-spectral interferences (matrix effects). The honey suspension was prepared by dissolution with ascorbic acid and HNO_3_ and homogenation in an ultrasonic bath. Isotope dilution and SA were then successfully used for Cr, Cd, Hg, and Pb quantification (additional examples of the use of SA will be presented in its specific section later on the text). Borno et al. (2015) used EC for elemental determination in low-density polyethylene by ETV-ICP OES. However, for quantification of the analytes in acrylonitrile butadiene styrene copolymer, the ETV heating program was not efficient at decomposing the organic matrix, which compromised the method's accuracy. As expected, EC is ineffective when differences in matrix between calibration standard solutions and samples become significant. Even when total digestion is employed for sample preparation, differences in physical and chemical properties, such as pH, viscosity, ionic strength, temperature, and surface tension can lead to poor accuracies in EC determinations (Bader, 1980).

An alternative to SA for compensating for matrix effects in elemental analyses is the use of MMC. In this method, the matrix of the calibration standard solutions mimic that of the samples. If the constitution of both calibration standards and samples closely match, negligible matrix effects are expected. MMC is normally accomplished by adding certified reference materials (CRMs) to the calibration standards in direct solid analyses, or by adding interference-causing concomitants to the standard solutions at concentrations close to the ones found in the sample solution. As an example, MMC with aqueous standard solutions prepared in 5% v/v formic acid was used for simultaneous elemental determination by ETV-ICP-MS in biological samples, which were previously prepared by formic acid solubilization (Tormen et al., 2012). In another work, Sánchez et al. (2016) used MMC to minimize matrix effects in ICP-MS analysis using a total sample consumption nebulizer. Inorganic standards prepared in a 1:1 (v/v) mix of ethanol and water were used for the direct analysis of a bioethanol sample diluted with water at the same proportion.

Many other works using MMC can be found in the literature. Exoskeleton of clams was analyzed by energy dispersive X-ray fluorescence (EDX) using five CRMs of bone ash, and bovine and caprine bone as calibration standards for MMC (Pessanha et al., 2018). Babos et al. (2018) also used MMC for Ca determination in mineral supplements for cattle by wavelength dispersive X-ray fluorescence (WD-XRF). In this case, a reference material of mineral supplements for cattle was diluted in solid phase with a Na_2_CO_3_:NaCl mixture. Rietig and Acker (2017) used MMC for trace element determination in a silicon sample previously digested with a mixture of HNO_3_ and HF. The standard solutions were matrix-matched by adding Si at levels similar to those expected for the sample solutions. In another work, Nunes et al. used filter paper embedded with reference solution as a solid standard for multielement analysis of botanical samples by ICP-MS coupled with laser ablation (LA; Nunes et al., 2016). The authors argue that filter paper presents similarities in composition with botanical materials, which makes it a good matrix-matching material for MMC.

Different from the procedure described by Amais et al. (2013) for MIP OES determinations, poor accuracies were obtained when using aqueous standard solutions and EC for elemental analysis of biodiesel samples dissolved in ethanol by both F AAS and flame atomic emission spectrometry (F AES). Alternatively, MMC using standard solutions prepared in washed biodiesel:ethanol at 1:20 v/v (Barros et al., 2012), or 1:10 v/v (Magalhães et al., 2014) resulted in adequate recoveries for F AES and F AAS determinations, respectively. The use of washed samples (i.e., biodiesel free of the analytes) for adjusting the viscosity of the standard solutions and matching it as close as possible to that of the samples has been proposed, in these cases, as an alternative to the use of mineral oils, which is recommended in the standard method ABNT NBR 15556 (2008).

Although efficient in some cases, MMC is difficult and cumbersome, as it becomes almost impossible to match the composition of more complex samples. Standard solutions usually have more than one property which is different from the samples', which frequently results in severe matrix effects despite the use of MMC (Cuadros-Rodríguez et al., 2001). Such differences can cause transport-related interferences during sample introduction in ICP OES, ICP-MS and F AAS. In laser-induced breakdown spectroscopy (LIBS), inhomogeneous interaction between laser and sample matrix causes variation in signal intensities, which frequently compromises repeatability and accuracy (Singh and Thakur, 2007). In addition, matrix differences between calibration standards and samples can result in different mechanisms of atomization, excitation and ionization taking place in plasmas and flames, or in different mechanisms of atomization in GF AAS. In many cases, and especially due to the complexity of some sample matrices, MMC is not capable of minimizing such effects. The traditional alternatives to correct for these interferences include IS and SA, as we will discuss in the following sections.

### Internal standardization (IS)

Internal standardization, previously known as reference element technique, was proposed by Gerlach and Schweitezer (1929) to correct for interferences in atomic spectrometry. It consists of adding a reference element called internal standard (IS) to all samples, calibrations standards and the analytical blank, and using the analyte-to-IS signal ratio for calibration. The IS species has a known concentration, and ideally should be affected by the same processes as the analyte during the instrumental measurement. Thus, if the IS signal behaves similarly to the analyte's, their ratio should be relatively constant, which then minimizes the effects of fluctuations due to nebulization, radiation source intensity, sample position, or other instrumental parameters on accuracy. Although generally ineffective for minimizing matrix effects, the IS method may be used to that end in certain instances. The IS calibration curve is built by plotting the analyte concentration on the *x*-axis (independent variable), with the analyte/IS signal ratio on the *y*-axis (dependent variable).

The IS method requires that signals from both the analyte and IS species are monitored simultaneously, or at least fast sequentially, during the analysis. Therefore, it has been mainly used in multi-element methods such as ICP OES, ICP-MS, and LIBS, with limited applications in F AAS and GF AAS (Welz and Sperling, 1998). In F AAS, the development of dual-channel instruments allowed the use of IS to correct for variations in the sample introduction system, as reviewed by Fernandes et al. (2003). It has also been employed in elemental determinations using multi-channel GF AAS (Takada and Nakano, 1981). The development of graphite furnace simultaneous atomic absorption spectrometry (commercially named SIM AAS) and fast-sequential flame instruments contributed to increasing the number of applications involving the IS method in atomic absorption spectrometry (Radziuk et al., 1999; Fernandes et al., 2002; Correia et al., 2004; Oliveira et al., 2004, 2005; Oliveira and Gomes Neto, 2007; Ferreira et al., 2008a,b; Miranda et al., 2010; Caldas and Sanches Filho, 2013; Barkonikos et al., 2014; Pasias et al., 2014; Pereira et al., 2014). More recently, such applications have been further extended with the combination of an efficient continuum source, a high-resolution Echelle polychromator, and solid-state detectors in high-resolution continuum source F AAS (HR-CS F AAS) and HR-CS GF AAS. Fast-sequential analysis can be easily carried out with these instruments (Raposo et al., 2008, 2012), and simultaneous determinations are also possible when analytical and IS lines appear in the same spectral window (Babos et al., 2016).

Choosing the ideal IS species usually follows two main criteria: (*i*) analyte (*A*) and IS signals should have similar intensities, with *A*/IS as close as possible to unity, and (*ii*) the physicochemical properties of both *A* and IS should be as similar as possible to insure they undergo the same processes during analysis (Barnett, 1968; Barnett et al., 1970; Fernandes et al., 2003). The most critical properties of an IS species will depend on the instrumental method used. According to Walsh (1992), the ideal IS should be present in a non-detectable concentration in the sample, should have low toxicity and low cost, should not form insoluble compounds, and should have an appropriate spectral line with no spectral interferences of its own. Table 2 shows some examples of applications using the IS method, and describes the selection criteria adopted for choosing a suitable IS species in each case.

**Table 2:** Internal standardization applied to SIM AAS, FS FAAS, ICP OES, and ICP-MS, and the selection criteria used for choosing the IS species.

**Instrumental method**	**Analyte**	**Internal standard**	**Selection criteria**	**Remarks**	**References**
SIM AAS	As and Se	Te	Comparison of physicochemical and kinetic properties	Correlation coefficients, intercepts and slopes were evaluated to verify similarities between analyte and IS. The best recoveries were obtained using Te as IS for As and Se	Barkonikos et al., 2014
SIM AAS	Cd and Pb	Ag	Comparison of physicochemical properties	Silver, Bi, and Tl were chosen as IS candidates. Correlation graphs between standard and IS, as well as evaluation of precision and accuracy were used to select Ag as the most appropriate IS	Correia et al., 2004
SIM AAS	Pb	Bi	Comparison of physicochemical properties	The efficiency of Bi as IS for Pb determination also was evaluated by means of correlation graphs (i.e., analyte vs. IS)	Fernandes et al., 2002; Oliveira and Gomes Neto, 2007
FS FAAS	Fe and Mn	Co to Mn and In to Fe	The IS species was chosen considering interferences caused to analytes lines and effects on flame composition	Corrections were performed using the following expression: *(AS_*C*_) = (AS)x*(${AS}_{RE}^{{}^{\circ}}$)/(AS_*RE*_) Where, *(AS)* is the analytical signal obtained during analyte determination, (${\mathrm{AS}}_{RE}^{{}^{\circ}}$) is the average of the analytical signals recorded for calibration solutions containing the IS, *(AS_*RE*_)* is the analytical signal recorded for the IS in the sample solution, and *(AS_*C*_)* is the corrected analytical signal	Ferreira et al., 2008b
FS FAAS	Cu	In	–	–	Ferreira et al., 2008a
FS FAAS	Cu	Ag	–	The slope and its standard deviation obtained by EC and IS was compared using Ag, Bi, Co, and Ni as IS. A two-sample *t*-test showed no significant differences between the calibration methods. The best accuracy was obtained using Ag as IS	Miranda et al., 2010
SIM AAS	Se	As	Comparison of physicochemical properties	The same behavior for analyte and IS absorbance signals was observed in different samples (correlation graph). This suggested the feasibility of using of As as IS for Se determination (or *vice-versa*)	Oliveira et al., 2004, 2005
SIM AAS	B	Ge	Comparison of physicochemical properties (for both volatile and stable oxides forms)	The use of IS increased the recovery and allowed a simple correction of errors during sample preparation and the heating process	Pasias et al., 2014
FS FAAS	Ca and Mg	Co	–	Internal standardization approach was enough to correct for transport effects in Ca and Mg determination	Pereira et al., 2014
SIM AAS	Pb	Bi	Comparison of physicochemical properties	The behavior of Pb was compared with Bi and Tl in different samples (urine, blood and placenta). Satisfactory signal correlation was observed when using Bi as IS	Radziuk et al., 1999
ICP OES	Mn	Sc	–	For calculating the analyte/IS ratio, plasma background emission was simultaneously subtracted	Schmidt and Slavln, 1982
ICP OES	Al, Ca, Fe, Na, Mg and Si	Cd as IS for Ca and Fe. Ga as IS for Al, Mg, and Si. Li as IS for Na	Evaluation of the behaviors of analytes and internal standards with different excitation potentials in various instrumental operating conditions	Three internal standards (Ga, Cd and Li) with different excitation potentials were evaluated. Analytical precision was improved using the IS method	Walsh, 1992
ICP OES	Al, Ag, Au, Cu, Fe, Ir, Mg, Mn, Ni, Pd, Rh and Zn	Y	Based on literature	Yttrium was introduced to eliminate effects caused by fluctuations in the plasma	Zhang et al., 1995
ICP OES	B and Ti	Y	–	Yttrium, Pd, Pt, and Sr were evaluated as IS to compensate for matrix effects. Yttrium was useful for signal correction in B and Ti determinations	Garavaglia et al., 2002
ICP OES	Sb	Cd	Comparison of physicochemical properties and wavelength proximity	The intensity ratio (*IR*) was calculated as *IR* = 100*ab*/*cd*. Where *a* is the intensity of Sb, *b* is the Cd solution mass (g), *c* is the solution volume after dilution (mL), and *d* is the signal intensity for Cd. The *IR* value for each calibration standard was plotted against the calculated concentration	Harmse and McCrindle, 2002
ICP OES	Ca, Mg, Mn, Fe, Zn and Cu	Y for Mn and Fe	–	Recoveries obtained with IS were better only for Mn and Fe	Sousa et al., 2005
ICP OES	Cd, Co, Cr and Mn	Y	–	Yttrium was used as IS to correct for transport effects caused by the residual salinity in the surfactant-rich phase	Bezerra et al., 2007
ICP OES	Ca, P, Mg, K and Na	Y	The internal standard species should present: concentration below the detection limit in the sample; high purity; and its analytical line should not cause spectral interferences on analytical lines	IS was used to compensate for transport effects	Santos et al., 2007
ICP OES	Ca, Cu, Fe, Mg, Mn, Na and P	Y	–	–	Souza et al., 2008
ICP OES	As	V	Based on literature	Cobalt, Bi, V and Y were evaluated as IS. Vanadium was efficient at minimizing sampling effects caused by sample heterogeneity (wine containing dissolved gases)	Mutic et al., 2011
ICP-MS/MS	B	Be	Comparison of physicochemical properties	Lithium, Be and Rh were evaluated as IS, and Be presented favorable characteristics due to similarities of its physicochemical properties with B	Amaral et al., 2016
ICP-MS	B	Be	Comparison of physicochemical properties (preliminary tests to choose the IS were carried out using the mass scanning mode of the data acquisition software)	More accurate results were obtained when the atomic mass of the IS species was close to the analyte's. The ionization energy was a secondary selection criterion	Vanhaecke et al., 1991
ICP-MS	Several elements^*^	One element was selected as analyte and other, with nearby atomic mass, was selected as IS^*^	–	–	Vanhaecke et al., 1992
ICP-MS	Al, As, B, Ba, Ca, Cd, Ce, Cs, Cu, Dy, Er, Eu, Fe, Gd, Ho, K, La, Li, Lu, Mn, Nd, Ni, P, Pb, Pr, Rb, S, Sm, Sn, Sr, Tb, Tm, Yb and Zn	In	–	Analytical signal depression due to the ethanol concentration in the sample (wine) was corrected by IS	Castiñeira et al., 2001
ICP-MS	Na, Mg, Al, Ca, Ti, V, Mn, Fe, Co, Ni, Cu, Zn,Ga, As, Mo, Cd, Sn, Sb, Ba and Pb	Sc as IS for Na, Mg, Al, Ca, Ti, V, Mn, Fe, and Co Rh as IS for Ni, Cu, Zn, Ga, As, Mo, Cd, Sn, Sb Ba, and Bi for Pb	Bi, Rh and Sc were chosen based on literature data	Relative standard deviations with different IS species were used as selection criteria	Xie et al., 2009

* *Not specified. No comments on the selection criteria*.

Bechlin et al. (2015) proposed Bi as a universal IS species for Pb determination. They demonstrated the efficiency of using the Pb/Bi pair in IS calibration for analyzing 34 assorted samples (household cleaning solutions, colored sugars, hard candies, mouthwash, orange, lemon, and grape juices, energy drink, tea, soft drinks, beer, vodka, sugar cane spirit, mineral water, vinegar, ethanol fuels, peanut, polyethylene terephthalate (PET) bottles, Maytenus ilicifolia, Peumus boldus, liquid fertilizer, solid fertilizer, shampoo and milk) by ICP OES, ICP-MS and ETV-ICP-MS. The analyte/IS combination was chosen based on similarity of physicochemical properties. According to the authors, Bi is a good IS for Pb in ICP OES analysis due to their similar enthalpies of vaporization, as well as their excitation energies, with 151 and 179.5 kJ/mol, and 5.55 and 5.71 eV, respectively. In ICP-MS, good accuracy was a result of similar molar masses and ionization energies, with 208.980 g/mol and 7.28 eV for Bi, and 207.977 g/mol and 7.41 eV for Pb. For ETV-ICP-MS, the performance of Bi as an adequate IS for Pb was attributed to the similarities between their vaporization enthalpies, atomic masses and ionization energies, as well as the dissociation energies of their chlorides and oxides. In another work, the authors have chosen Co (313.221 nm) as IS for Ni (313.410 nm) determination by direct solid sampling and HR-CS GF AAS, based on physicochemical properties criteria (atomic mass, melting point, boiling point, enthalpy of fusion and formation of gaseous atoms, and oxide dissociation energy). As discussed earlier, the use of IS in HR-CS GF AAS determinations was possible because both Co and Ni absorption lines occur within the same spectral window. Because of their similar characteristics, using Co as an IS for Ni contributed to minimizing matrix effects and to enabling the use of aqueous calibration standards (Babos et al., 2016).

According to Ramsey and Thompson (1985), principal components analysis (PCA) is a useful method for selecting the ideal (or as close to ideal as possible) IS species for different analytes. Grotti et al. (2005) and Grotti and Frache (2003) were based on this premise when they used a systematic procedure involving PCA to select IS species. Emission lines from analytes and potential IS species were evaluated, which formed clusters in a PCA scores plot according to their empirical behavior. In another work, multivariate optimization and exploratory analysis were used by Froes-Silva et al. (2015) to select IS species for elemental determination in complex matrices (i.e., synthetic reference solutions simulating several beverages, pharmaceutical products and foods) by ICP OES. According to the authors, elements in this study were correlated according to their physicochemical properties.

Other aspect emphasized in the literature about the selection of an IS species in ICP OES determinations is associated with the requirement for matching atomic or ionic lines, i.e., IS atomic lines should be paired with analyte atomic lines and, analogously, IS ionic lines should be used to correct for fluctuations in analyte ionic lines. In this sense, reports are ambiguous. Myers and Tracy (1983) showed that a single IS was capable of improving analytical performance independent of the analytical lines used. On the other hand, Aguirre et al. (2010) have shown that the Zn atomic line at 213.857 nm was the best IS for analytes with atomic emission lines, while the Zn ionic line at 202.548 nm was the best for analytes with ionic emission lines.

Chiweshe et al. (2016) evaluated different IS species for precious metals quantification in geological CRMs solubilized by digestion or fusion. The aim of this study was to evaluate some criteria for choosing an IS, and decide whether Co (although Y, Sc, and La were also studied) could be used as IS for all precious metal analytes surveyed. The authors compared parameters such as the atomic/ionic line pairing requirement, and similarities in ionization and/or excitation energies. According to them, even after using these properties and multivariate methods, the results were inconclusive to decide on the best IS. This study illustrates one of the main limitations of the IS method, i.e., identifying an IS species is never trivial. The authors ended up choosing Sc as IS based on adequate recoveries (>99%) from determination of precious metals in a liquid reference material.

Da Silva et al. (2007) used the IS method to compensate for matrix effects in wine-vinegar analysis by ICP OES. In this work, they used a cross-flow nebulizer combined with a double-path spray chamber, and a cone spray associated with a cyclonic spray chamber. The vinegar samples were directly aspirated into the plasma after simple dilution with water. All standard solutions (Al, Ba, Ca, Cu, K, Mg, Mn and Zn) were matched with 3% v/v acetic acid, and Sc and Y were evaluated as IS species. Even though the ionization energy for Sc (6.56 eV) is different from many of the analytes (Al−5.98 eV; Ba−5.21 eV; Ca−6.11 eV, Cu−7.72 eV, K−4.34 eV, Mg−7.64 eV, Mn−7.43 eV, and Zn−9.39 eV), the best recoveries were obtained with this IS under robust conditions and using a cone spray nebulizer and cyclonic spray chamber.

Similar to ICP OES, the selection of IS species in ICP-MS is poorly understood. Some authors use physicochemical properties as a criterion for choosing an IS species (Vanhaecke et al., 1991; Amaral et al., 2016), while others provide no discussion on the reasons to use a given IS (Castiñeira et al., 2001). According to a study by Finley-Jones et al. (2008), which involved multivariate analysis, proximity between the atomic masses of IS and analyte is the most appropriated criteria for selecting an IS species in time-of-flight-based ICP-MS [ICP-(TOF)MS]. However, as frequently is the case in IS, there were several exceptions to this general rule. Recently, Olesik and Jiao (2017) observed that a single IS species was capable of correcting for matrix effects on analytes of different atomic masses (at low, mid, and high range) using an ELAN 6000 ICP-MS. They have since performed measurements using a Thermo Element 2 and a PerkinElmer NexION 350D, and the results should be reported in future publications.

Salazar et al. (2011) reported the use of Sc as the best IS species for 18 out of 28 analyte isotopes evaluated. The authors argue that Sc is a good IS in ICP-MS due to the proximity of its mass and/or first ionization energy with those of the analytes. However, as it is common in IS studies, there were some exceptions: ^115^In^+^ was the best IS for ^50^V^+^, and ^103^Rh^+^ was the best IS for ^63^Cu^+^. Barros et al. (2018) also observed divergences when studying the criteria for selecting the best IS in ICP-MS. They observed improvement in recoveries for ^9^Be when ^7^Li was used as IS, which may be attributed to the similar mass-to-charge ratios (*m/z*). However, in the same study, they saw significant improvement in ^27^Al recoveries using ^193^Ir as IS, which is difficult to explain considering the different physicochemical properties and *m/z* values of these elements.

Another strategy for selecting an IS species is to adopt a major constituent of either the sample or the plasma. Scheffler et al. (2016, 2017), for example, used an Ar line at 415.859 nm as IS to compensate for plasma loading effects in elemental determination by ETV-ICP OES. Unfortunately, this strategy is not applicable to correct for fluctuations due to aerosol generation and transport, as it only relates to effects on the Ar plasma. According to Scheffler and Pozebon (2013), while Ga, In and Y were employed successfully as IS, the 415.859 nm Ar line was ineffective at correcting for matrix effects on Ba, Cd, Co, Cu, Cr, Mn, Ni, Pb, Sr, and V determination by ICP OES. On the other hand, selecting an IS species which is a major constituent of the sample has been fairly successful in LIBS determinations. Lucena et al. (1998), for example, used Ti as IS to compensate for pulse-by-pulse variation and matrix effects in V determination in a catalyst of TiO_2_ supported on silica (2TiO_2_ – SiO_2_). In another work, Na was used as IS for Ca determination in biochar-based fertilizer for minimizing differences between the sample and the calibration standards, which were built using eucalyptus biochar as a matrix match (Morais et al., 2017). In this same context, Fe has been used as IS to determine several elements in complex-matrix samples such as soil (Kwak et al., 2009; Kim et al., 2013), steam generator tubes (Whitehouse et al., 2001), and industrial alloys (Latkoczy and Ghislain, 2006). In all cases, IS led to improvement in the analytical calibration curve linearity. Other major elements have also been used as IS in LIBS for several applications: Cu for the analysis of bronze samples submerged in sea water (De Giacomo et al., 2005), Ni for the analysis of nickel-based alloys (Gupta et al., 2011), Al for aluminum-based alloys (Mohamed, 2008), and O for phosphor synthetized samples (Unnikrishnan et al., 2013) and for spinach and rice samples contaminated with pesticides (Kim G. et al., 2012).

As an example of a non-successful application of this strategy, Štěpánková et al. (2013) evaluated the use of Ca as IS for the analysis of urinary calculi by LIBS. According to the authors, Ca concentrations were not homogeneous throughout the samples due to the different crystalline phases, which compromised the efficiency of the IS method. In addition to the inhomogeneity issue and the difficulty in finding an element which presents a constant concentration throughout the sample as described by Štěpánková et al., another drawback of internal standardization with a major element is the resulting poor sensitivities of the analytical calibration curves. Because of the major element's high signals, the analyte/IS ratio used for calibration may become too low.

Even though IS has been successfully used to correct for matrix effects in several applications involving different analytes and instrumental methods, the mechanisms by which it works must be better understood, especially considering the diverging reports in the literature on the criteria to select an effective IS species. In this context, strategies such as similarities in physicochemical properties between analyte and IS, multivariate analysis, use of major constituents, and the use of validation results have been the main criteria for IS selection. Although effective in many applications, a better rationalization for IS selection and application would be highly useful in routine analyses.

### The standard additions method (SA)

**Figure 1:**
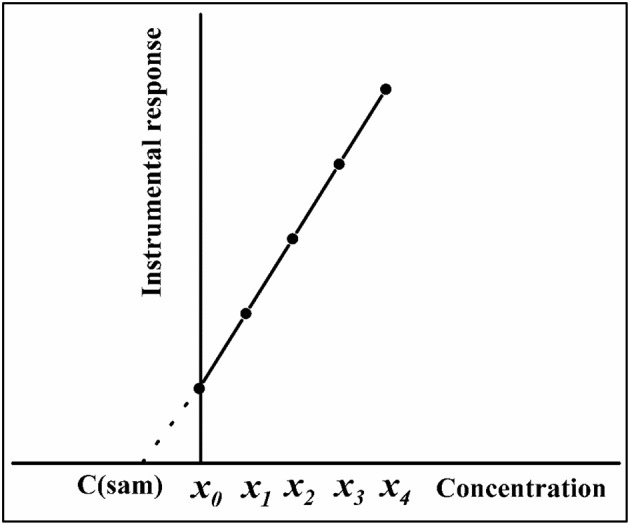
SA calibration plot using four addition points (*x*_1_-*x*_4_). Extrapolation is represented by a broken line, and *x*_0_ is the sample with no standard addition.

According to Kelly et al. (2011), the SA method, or standard additions calibration (SAC, also previously known as addition method of analysis), was described for the first time in 1937, in a book on polarography by Hans Hohn. However, it was used in XRF and atomic spectrometry only 20 years later. In SA, the calibration solutions are prepared by adding increasing concentrations of the analyte to the sample, and the sample solution itself is used as solvent to minimize matrix effects. The analyte concentration added to the sample solutions (or the volume of the standard solution added to the sample) is plotted as independent variable on the *x*-axis, with instrument response as the dependent variable on the *y*-axis. Similar to EC and IS, the calibration function is estimated by least squares fitting. Figure 1 shows an example of a SA plot using five calibration points: no analyte added (*x*_0_), and four analyte additions with increasing concentrations (*x*_1_-*x*_4_). According to the IUPAC, the analyte should be added in equimolar amounts, i.e., *x*_1_ = *x*_0_; *x*_2_ = 2*x*_1_; *x*_3_ = 3*x*_1_, and *x*_4_ = 4*x*_1_ (Currie, 1998). In general, the unknown analyte concentration in the sample [*C*_(*sam*)_] can be obtained by geometrical extrapolation of the *x*-axis, at *y* = 0 [i.e., *C*_(*sam*)_ = -*a*/*b*, where *a* is the intercept of the regression line, and *b* is the slope estimated by least squares fitting] (Currie, 1998; Kelly et al., 2011; Gonçalves et al., 2016a). It can also be determined by the interpolation method, which consists of interpolating *C*_(*sam*)_as twice the signal generated by the sample (Andrade et al., 2013). A minor difficulty associated with the application of the SA method is the determination of the error in the measurement. Gonçalves et al. (2016a) proposed a reversed-axis approach, based on least-squares regression using Microsoft Excel, in which the analyte concentrations added to the sample were plotted on the *y*-axis, and instrument response was plotted on the *x*-axis. When instrument response is zero, C_(sam)_ can be determined from the *y*-axis intercept [*C*_(*sam*)_ = *a*], and the standard deviation of analyte concentration in the sample (*s*_*c*_) is automatically calculated by the software as the standard deviation of the intercept (*s*_*a*_).

Several papers have applied the extrapolation strategy for SA, but according to Andrade et al. (2013), the use of interpolation increases accuracy and precision because the central part of the regression line (where the confidence interval of the regression is narrow) is used in the calculations. Additional details on the mathematical description and theory of the SA method can be found in the literature in works by Kolthoff and Lingane (1939), Bader (1980), Currie (1998), Bergfeld et al. (2005), Brown et al. (2007, 2011, 2017), Brown (2009), Kelly et al. (2011), Brown and Gillam (2012a,b), Brown and Mustoe (2014), Gonçalves et al. (2016a), Andersen (2017), among others.

There are several strategies to perform SA. In voltammetric analysis, frequently only two calibration points are used, with solution 1 containing the sample with no standard added, and solution 2 consisting of the sample with standard addition (Kolthoff and Lingane, 1939; Kelly et al., 2011). In these cases, a mathematical equation, rather than graphical extrapolation, has been used to determine the analyte concentration in the sample. In spectrometric analysis, typically five or six calibration points and graphical extrapolation have been used, since the 1950s, for calculating the analyte concentration in the sample (Kelly et al., 2011).

Bader (1980) described a systematic approach to SA including five different strategies to perform this calibration method when analyte concentration and instrument response present a linear relationship. As discussed by the author, a different mathematical equation is used to calculate the unknown analyte concentration in the sample for each of the five proposed strategies. The mathematical equations use values for the intercept, slope, the concentration of the standard solution, and the volumes of both standard solution and sample. Kelly et al. (2008) revised the equations for all five SA strategies proposed by Bader considering a gravimetric approach. Masses rather than volumes were used in this case, and according to the authors, such approach makes the SA method suitable not only for liquid solutions, but also for solid samples.

When several solutions containing the same amount of sample and different amounts of standard solution are prepared separately, SA is known as conventional standard additions calibration (C-SAC). The first of these solutions contains only the sample, while increasing concentrations of the analyte are added the other solutions (Figure 1). In sequential standard additions calibration (S-SAC), different amounts of standard are added to a single flask containing the sample. In S-SAC, the original sample in a container is analyzed and the instrument response is recorded (Brown et al., 2007). Then, a certain amount of standard solution is added to the same container and the analytical signal is recorded once again. The procedure is repeated, with additional volumes of the standard solution sequentially added to the same container and the analytical signals recorded in each case. The SA calibration plot is then built with analyte concentrations (or standard volumes) added to the sample (*x*-axis) vs. instrument response (*y*-axis). Another application for S-SAC was proposed by Brown et al. (2017), in which the final volume of the mixture is used to determine the concentration of relevant impurities in a blank matrix of gas. The constant final volume strategy is only applicable to gas samples, but the extrapolation method used with it is the same as the one used with variable-final-volume S-SAC.

Brown (2009, 2011), Brown and Gillam (2012a,b) and Brown et al. (2007, 2011) evaluated the differences between C-SAC and S-SAC regarding precision, use of extrapolation or interpolation, and homoscedasticity or heteroscedasticity. The authors have shown that S-SAC can replace C-SAC if an adequate quantity of standard solution is added to the sample, and if the appropriated equation (which was proposed by the authors) is used to determine the analyte concentration in the sample. The main advantages of S-SAC when compared to C-SAC is its simplicity, lower costs, higher sample throughput, and generation of less residues (Brown et al., 2007; Brown, 2009, 2011). However, the application of S-SAC may be limited to non-destructive instrumental methods, such as voltammetry and UV-visible spectrophotometry. So far, there is no report in the literature of the use of S-SAC in spectrometric instrumental methods. However, it may be easily applied if the volume of sample consumed during the analysis is small, and the volume of standard solution added in each step is negligible in comparison with the total volume of the sample solution. For example, 10.0 μL of standard solution is added to 25.0 mL of sample in each standard addition step in a GF AAS or a tungsten coil atomic emission spectrometry (WCAES) determination (which typically consume 10–25 μL of sample per run). In this case, because the volumes of standard solution added and solution mixture consumed during the analysis have no significant effect on the final solution volume, S-SAC can be successfully applied. It is important to note, though, that S-SAC is an irreversible method, i.e., once the standard solution is added, there is no way to re-run a sample to check a previously recorded signal.

Other approaches to SA include, for example, the bracket standard additions method described by Abbyad et al. (2001) to improve precision in ICP-MS. It consists of recording the analytical signal before and after adding the standard solution to the sample, and using the mean of the two measurements in specific equations to compensate for instrument drift. The authors also evaluated different levels of standard addition and showed that similar precisions are obtained when the analyte concentration in the standard solution is the same as, or 7- to 50-fold higher than that of the sample. In another work, Boto and Murphy (2005) proposed modifications to the SA equation to overcome issues associated with its application to samples containing very high concentrations of analyte. Their method was used in XRF and may be applied to other instrumental analytical techniques.

Before the revision of Bader's equations by Kelly et al. (2008), Christopher et al. (2005) had introduced the gravimetric approach to the SA method. In addition to its compatibility to solid samples as discussed earlier, the authors pointed out that the gravimetric approach may also improve accuracy when compared to the volumetric approach. Internal standardization was combined with the gravimetric approach to overcome the inconvenience associated with sample solution dilution when the standard is added. The IS species and the analyte standard were gravimetrically added to each flask containing the sample (usually a certified reference material), with concentrations varying from none to a high addition level. According to the authors, the main advantage of this approach is that quantitative adjustment of volume is not necessary, as the IS can correct for dilution effects. Furthermore, if samples are homogenous and have identical matrix, it is not necessary to build a calibration curve for each sample, and the analyte concentration can be determined from the slope of a curve built with the certified reference material. In this case, the calibration plot contains the mass of analyte added per gram of sample on the *x*-axis, and the analyte/IS signal ratio, multiplied by mass of IS per gram of sample, on the *y*-axis.

Zhu and Chiba (2012) proposed combining the SA gravimetric approach with internal standardization using a single standard addition point for elemental analysis by ICP-MS. The analyte concentration in the sample is determined by using the appropriate equation and considering the signal and mass of analyte and IS in both the standard solutions and the sample. A different mathematical equation is proposed if the mass of the IS species is not known. As reported by the authors, any element can be used as IS. However, because there is no requirement for similar physicochemical properties nor close *m/z* values for analyte and IS, the element selected as an IS must present a high signal intensity, stable concentration, and have no signal overlap with the analyte. This strategy was also used by Gao et al. (2015a,b) to determine As and Sb in seawater and natural water by ICP-MS. As one would expect, the one-point standard addition calibration method is simpler, less expensive and faster than the traditional multi-level standard additions (Andrade et al., 2013). However, similar to EC, the more calibration points used, the more precise and accurate the results (Miller and Miller, 1984). Table 3 shows some selected applications using the SA method applied to instrumental spectrometric techniques, including the combination of SA and IS, which is frequently used in LIBS.

**Table 3:** Selected applications of the SA method associated with instrumental spectroanalytical techniques.

**Sample**	**Analyte**	**Instrumental method**	**Remarks**	**References**
Copper-based materials	Ni	F AAS	Conventional SA was used with two standard addition points	Carril et al., 1996
Biodiesel	S	XRF	Gravimetric SA was used. The value for *[(m_*x*_+m_*s*_)/m_*x*_]IR_*x*_* was plotted on the *y*-axis, while *(m_*s*_ x c_*s*_)/m_*x*_* was plotted and on the *x*-axis. Where *m_*s*_* and *m_*x*_* are the mass of the standard solution and the sample, respectively; *IR_*x*_* is the instrumental response for the analyte in the sample, and *c_*s*_* is the concentration of the standard solution. The unknown analyte concentration (μg/g) was determined by extrapolation	Barker et al., 2008
Ore sample	U	LIBS	Conventional SA was used with five standard addition points. Additionally, a Ca line was used for internal standardization. The U/Ca signal ratio was plotted on the *y*-axis, with analyte concentration on the *x*-axis	Kim Y. S. et al., 2012
Soil	Pb	LIBS	Conventional SA was used with five standard addition points. SA and background removal were combined. The background was removed by discrete wavelet transform	Yi et al., 2016
Dairy milk and protein	Ca	LIBS	Conventional SA with three standard addition points was used. The unknown analyte concentration was determined by extrapolation. Additionally, K, C, K, Mg and Na lines were used for internal standardization. The analyte/IS signal ratio was plotted on the *y*-axis, with analyte concentration on the *x*-axis	Alfarraj et al., 2018
Peppermint tea	Mn and Ba	LIBS	Conventional SA with four standard addition points was used. The unknown analyte concentration was determined by extrapolation. The SA method was evaluated without IS, as well as using the background or Sr for internal standardization. The analyte/Sr signal ratio was plotted on the *y*-axis, with analyte concentration on the *x*-axis. Strontium was used as IS because of its constant concentration in the samples	Zivkovic et al., 2018
Silicon carbide powders	Al, Fe, Mn, Ti and V	SA ICP OES^*^	Conventional SA with four standard addition points was used. Internal standardization with Y (200 μg g^−1^ added) was used in combination with SA	Kiera et al., 2008

* *SA, spark ablation*.

The main advantage associated with the SA method is that the sample itself is used to prepare the calibration solutions, consequently matrix effects are corrected and no previous knowledge of the sample matrix is required (Kelly et al., 2011). The SA method has been shown efficient at correcting for rotational effects, which involve non-analyte constituents of the sample and their impact on the calibration curve slope. On the other hand, the SA method is not suitable for correcting for translational effects, which are related to the background signal and only affect the calibration curve intercept (Thompson, 2009; Andrade et al., 2013; Andersen, 2017). In this case, concomitant substances in the matrix cause systematic variation of the analytical signal, compromising accuracy and precision (Thompson, 2009; Andrade et al., 2013). The main drawback associated with the SA method is the requirement for building a calibration curve for each sample. This issue becomes especially critical when a large amount of samples has to be analyzed, as SA's sample throughput is considerably lower than EC, MMC, and IS. Additionally, the SA method may become ineffective, due to large experimental error, when the chemical form of the analyte is different in the sample and standard solution added, when the analyte is present at very high concentrations in the sample, and depending on the ratio between the concentration of analyte in the standard solution added and in the sample (Boto and Murphy, 2005; Andrade et al., 2013).

## Non-traditional calibration methods applied to ICP-MS

Several non-instrumental strategies have been described to minimize spectral and non-spectral interferences in ICP-MS determinations. Mathematical correction equations are an example of a data-processing approach used prior to calibration to minimize spectral interferences. By knowing the natural abundance of each isotope of an interfering element and recording its signal intensity at an alternative *m/z*, one can easily subtract the interfering signal at the analyte's *m/z* (Goossens et al., 1994; Neubauer, 2010; Thomas, 2013). Although efficient in some specific cases involving isobaric monoatomic interfering species, mathematical correction equations present several drawbacks. They perform poorly when high concentrations of the interfering species are present. On the other hand, if there is no interference, applying a correction equation will cause overcorrection, which in many cases results in obviously wrong negative analyte concentrations. In addition, increasingly more complex equations are required if the alternative isotope used for signal correction suffers itself from spectral interferences.

As an example of a strategy to minimize non-spectral interferences in ICP-MS, naturally-occurring polyatomic ions such as ArH^+^, ${\text{N}}_{2}^{+}$, ArO^+^, and ${\text{Ar}}_{2}^{+}$ have been employed as internal standard species to correct for matrix effects and signal drift (Beauchemin et al., 1987). These ions were used, for example, to minimize signal bias in As, Co, Mg and Mn determinations, and the ${\text{Ar}}_{2}^{+}$ species was applied to correct for transport and matrix effects in the direct analysis of solids by ETV-ICP-MS (Grégoire et al., 1994; Chen and Houk, 1995; Vanhecke et al., 1995).

More recently, four non-conventional calibration strategies have been reported to improve accuracy in quadrupole-based ICP-MS analyses. The IFS method is similar to the previously discussed strategy of using naturally-occurring polyatomic ions to correct for signal bias. However, it is used to minimize spectral interferences, and therefore, works differently from a traditional IS method. The other three strategies, SDA, MICal, and MSC, are used to minimize non-spectral interferences, and are based on a matrix-matching approach which uses only two calibration solutions per sample. These novel strategies will be discussed in detail in the following sections.

### Theory and mathematical description of some non-traditional calibration methods

#### The interference standard method (IFS)

**Figure 2:**
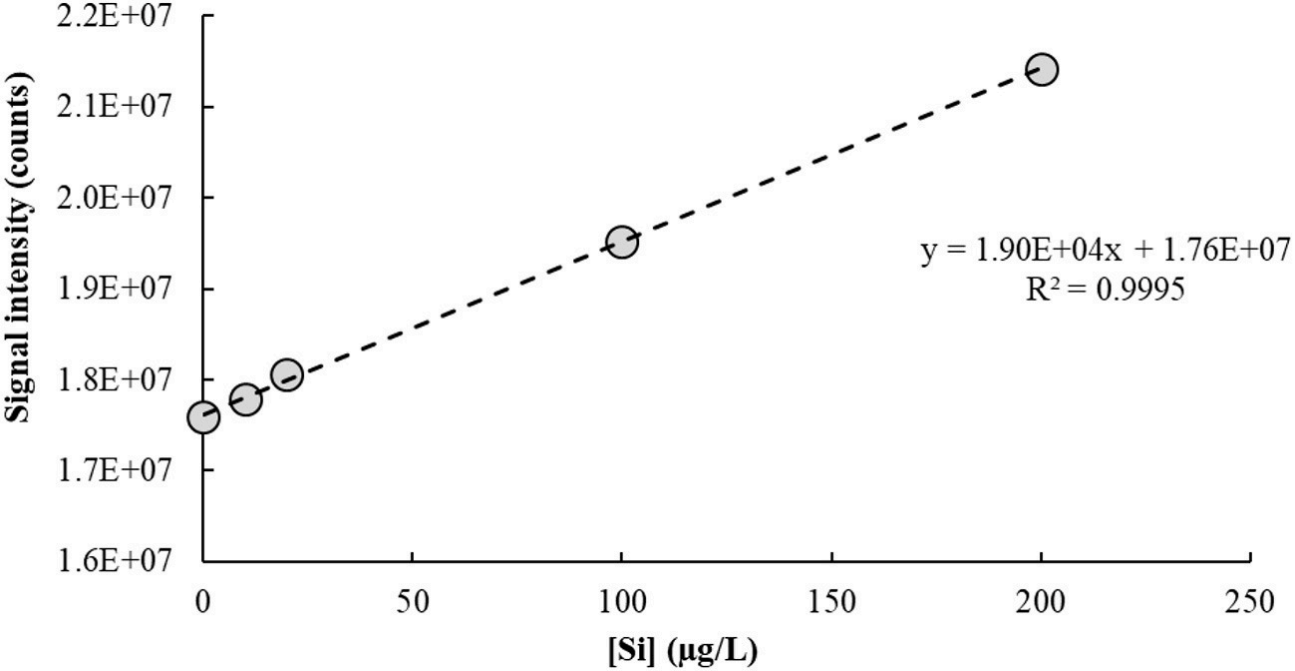
Calibration plot used to determine Si in a 1% v/v HNO_3_ matrix by ICP-QMS (*m/z* = 28). No collision/reaction gas was used.

**Figure 3:**
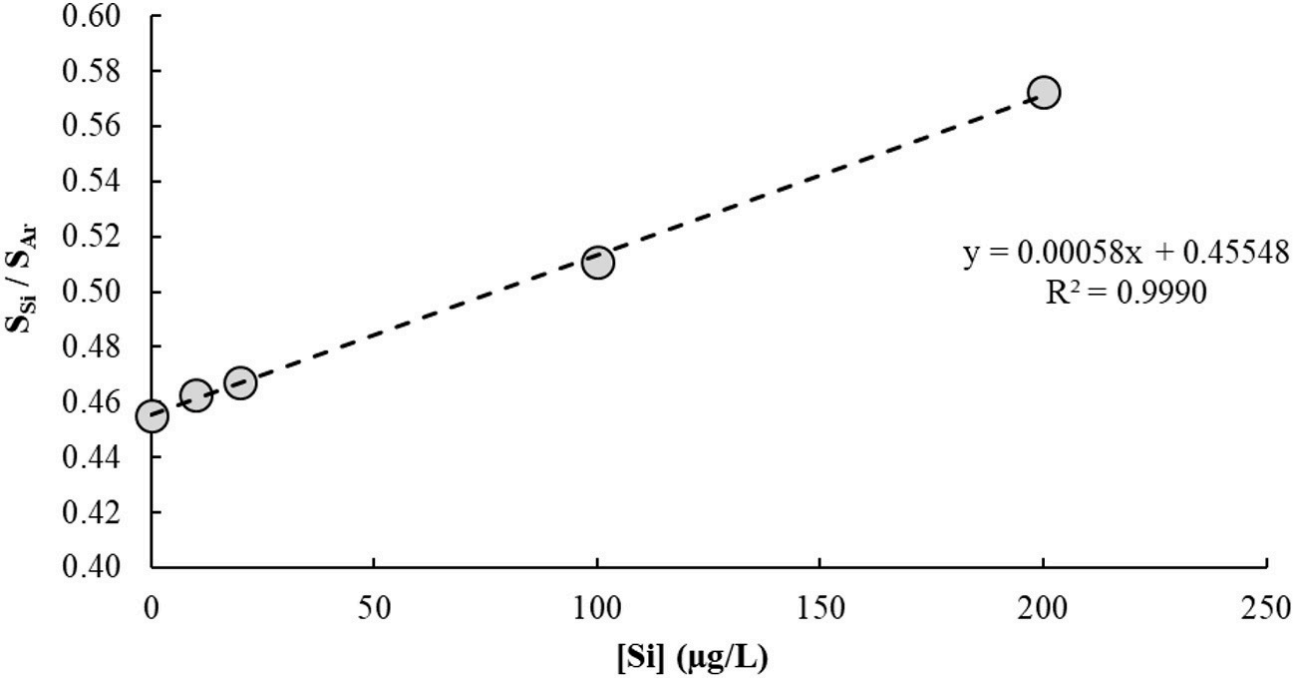
Calibration plot used to determine Si in a 1% v/v HNO_3_ matrix by ICP-QMS (*m/z* = 28) using the IFS method. The ^38^Ar^+^ ion was used as IFS species, and the analyte-to-IFS signal ratio was plotted on the *y*-axis. No collision/reaction gas was used.

The IFS method is a calibration strategy proposed to overcome polyatomic interferences in ICP-QMS. It relies on the hypothesis that interfering ions and naturally-occurring IFS species such as ^36^Ar^+^, ^36^ArH^+^, and ^38^Ar^+^ present similar behaviors in the plasma (Donati et al., 2011). Additional evidence of this principle has been provided in a recent work using high-resolution sector field ICP-MS measurements (Amais et al., 2014). Different from a conventional internal standardization method, the IFS strategy considers correcting variations in the interfering signal rather than in the analytical signal. Therefore, signal bias caused by ions and polyatomic species naturally occurring in the plasma, or formed by interactions between these and the sample solution, can be minimized by using the signal ratio between analyte and IFS species during calibration (Donati et al., 2011). It is important to note that the relatively low resolution of an ICP-QMS is not enough to separate analyte and interfering signals, so both are divided by the IFS signal during data processing. The main premise of the IFS method is that because interfering polyatomic species and IFS species behave similarly, their signal ratio will be relatively constant, which can then minimize biases caused on the analytical signal by variations in the unresolved isobaric interfering signal. Thus, a typical IFS calibration plot has the ratio of unresolved analyte + interfering signal/IFS signal on the *y*-axis (dependent variable), and analyte concentration on the *x*-axis (independent variable). Compare, for example, Figures 2 and 3, which represent the calibration plots for Si determination by ICP-QMS with and without applying the IFS method. Using the regression equation in Figure 2 to calculate the analyte concentration in a sample spiked with 10.0 μg/L of Si results in a negative value: −10.7 ± 1.5 μg/L (*n* = 3). On the other hand, the regression equation based on the IFS method (Figure 3) provides an accurate result of 9.8 ± 1.5 μg/L of Si (*n* = 3). This example highlights how severe the effect from the ^14^${\text{N}}_{2}^{+}$ ion can be on Si determination, at the *m/z* 28, when no signal correction nor other interference elimination strategy is used in ICP-QMS. It also shows the efficiency of the IFS method at minimizing spectral interferences and improving accuracy. As demonstrated in previous works (Donati et al., 2011, 2012a; Amais et al., 2014), the ^38^Ar^+^ IFS ion has a similar behavior to the ^28^${\text{N}}_{2}^{+}$ ion, and when their signal ratio is used for calibration, the interfering effect is significantly reduced.

These results can be further understood by analyzing Equations (1) and (2), where *R* is the calculated analyte recovery (%), *S*_*I*_ is the interfering ion signal collected during calibration, *V*_*I*_ is percent difference between interfering ion signal values recorded for the sample and its corresponding standard solution (i.e., it represents how much the interfering ion signal has varied from the time the calibration standards were run to when the sample was analyzed), *S*_*A*_ is the analytical signal, and *V*_*IFS*_ is the percent difference between IFS signal values recorded for the sample and its corresponding standard solution (i.e., similar to *V*_*I*_).

(1) $$\begin{array}{r} R\left(\%\right)=\left(1+\frac{S_{I}V_{I}}{S_{A}}\right)\cdot 100 \end{array}$$

(2) $$\begin{array}{r} R\left(\%\right)=\left[\frac{1}{1+V_{IFS}}+\frac{S_{I}}{S_{A}}\left(\frac{V_{I}-V_{IFS}}{1+V_{IFS}}\right)\right]\cdot 100 \end{array}$$

From Equation (1), one can infer that the larger the variation in the interfering ion signal (*V*_*I*_) and the larger the *S*_*I*_/*S*_*A*_ ratio, the worse the analyte recoveries (Donati et al., 2011; Amais et al., 2014). In the example of Si determination discussed earlier, *V*_*I*_ might have been negative, i.e., the interfering ion signal was lower when the sample was analyzed than when its corresponding standard solution was run, which resulted in a negative *R (%)*. Considering that N_2_ gas is the major component of air, and it is dragged into the Ar plasma during ICP operation, it is also reasonable to assume the ^14^${\text{N}}_{2}^{+}$ signal (*S*_*I*_) was significantly higher than the ^28^Si^+^ signal (*S*_*A*_) during the analysis, which also contributed to the poor recovery. Alternatively, when the IFS method was applied, it is reasonable to assume that *V*_*I*_ and *V*_*IFS*_ were relatively similar (Equation 2), which reduced the impact of the large *S*_*I*_/*S*_*A*_ ratio on analyte recovery (i.e., *S*_*I*_/*S*_*A*_ is multiplied by a number close to zero), and provided a more accurate concentration value. Similar results and related discussions were presented in a recent work employing high-resolution ICP-MS measurements, which also described the fundamentals of the IFS method in more detail (Amais et al., 2014).

#### Standard dilution analysis (SDA)

SDA is a calibration method that may be applied to any instrumental technique which accepts liquid samples and is capable of monitoring multiple analytical signals either simultaneously or fast sequentially (Jones et al., 2015; Gonçalves et al., 2016b; Virgilio et al., 2016a,b). It is an example of analytical strategy to minimize non-spectral interferences, as it combines the traditional methods of IS and SA. Thus, SDA is able to simultaneously correct for matrix effects and signal fluctuations due to changes in sample size, orientation, or instrumental parameters (Jones et al., 2015).

**Figure 4:**
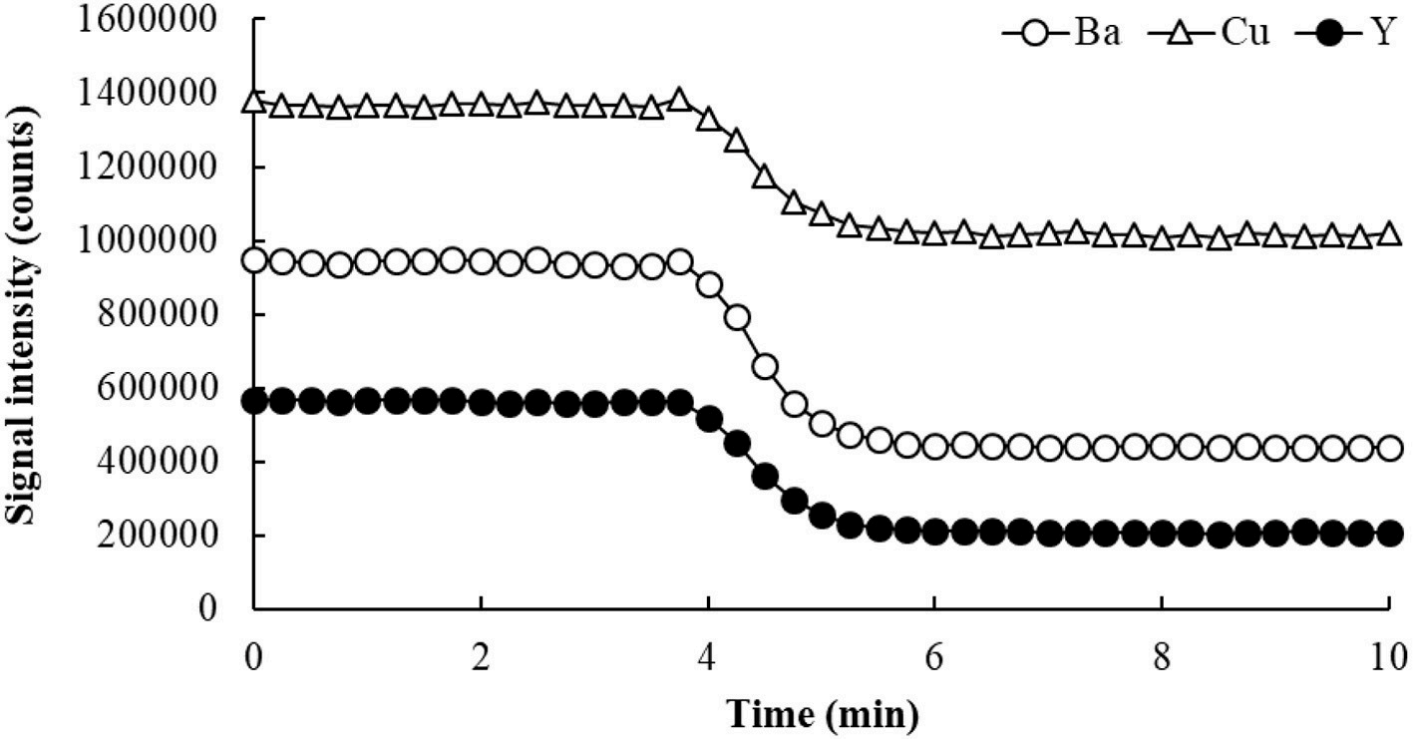
SDA plot for the determination of Ba and Cu in Oyster Tissue (NIST 1566b) by ICP-MS. Yttrium was used as internal standard, and He flowing at 3.5 mL/min was used in the collision/reaction cell.

Applying the SDA method involves the continuous monitoring of both analytes and an internal standard species, as two calibration solutions (S1 and S2) are mixed in a single container. These solutions contain the same amount of sample, so no matrix effect is expected. S1 has a 1:1 proportion of sample and a standard solution containing the analytes and the internal standard (IS), while S2 has the same 1:1 mixture of sample and blank. Data is initially collected, in a time-resolved manner, as S1 is introduced into the ICP-MS. When S2 is poured into the same container with S1, the sample matrix concentration is not affected (because both S1 and S2 contain the same amount of sample), but the standards and the IS are gradually diluted as the two solutions mix (hence SDA). Both analytical and IS signals slowly drop as a result, creating a negative slope on the signal intensity vs. time plot. In the negative slope region, known as the SDA region (Figure 4), each point represents a different concentration of analyte and IS. Thus, similarly to a conventional EC plot, these “*calibration points*” can be used to determine the analyte concentration in the sample. Only in this case, several calibration points are potentially available, as signals are collected typically every second (Virgilio et al., 2016a).

**Figure 5:**
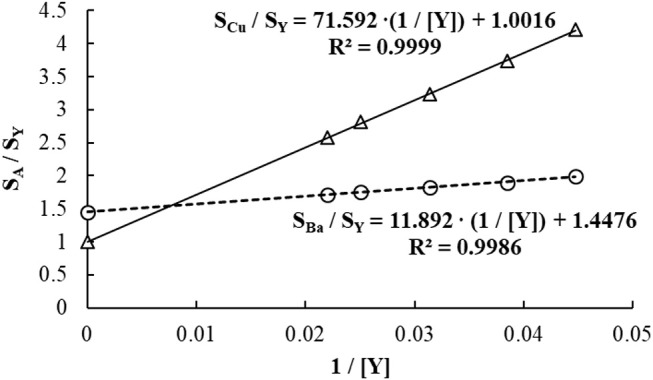
Calibration plots created from the SDA region (negative slopes) in Figure 4. The concentrations of Ba, Cu, and Y added to S1 were 50 μg/L each.

The SDA calibration curve is built by plotting the analyte-to-IS signal ratio (*S*_*A*_*/S*_*IS*_) on the *y*-axis, and the reciprocal of the IS concentration (1/C_IS_) on the *x*-axis (Jones et al., 2015; Virgilio et al., 2016b). Figures 4 and 5 show an example of SDA application to determine Ba and Cu in a standard reference material of Oyster Tissue (NIST 1566b). Values for *C*_*IS*_ are calculated from the known IS concentration added to S1. In this case, any point on the plateau preceding the negative slope in Figure 4 corresponds to that concentration (50 μg/L in this example), and each subsequent point in the SDA region has a proportionally lower concentration. According to Equation (3) (Jones et al., 2015; Gonçalves et al., 2016b; Virgilio et al., 2016a,b), the analyte concentration in the sample (*C(sam)*_*A*_) can be found from the slope and intercept of the SDA calibration plot, which are represented in Equations (4–6).

(3) $$\begin{array}{r} \frac{S_{A}}{S_{IS}}=\;\frac{m_{A}\left[C{\left(sam\right)}_{A}+C{\left(std\right)}_{A}\right]}{m_{IS}\;C_{IS}}=\;\frac{m_{A}C{\left(sam\right)}_{A}}{m_{IS}\;C_{IS}}+\;\frac{m_{A}C{\left(std\right)}_{A}}{m_{IS}\;C_{IS}} \end{array}$$

(4) $$\begin{array}{r} Slope=\;\frac{m_{A}C{\left(sam\right)}_{A}}{m_{IS}} \end{array}$$

(5) $$\begin{array}{r} Intercept=\;\frac{m_{A}C{\left(std\right)}_{A}}{m_{IS}\;C_{IS}} \end{array}$$

(6) $$\begin{array}{r} C{\left(sam\right)}_{A}=\frac{Slope}{Intercept}\cdot \frac{C{\left(std\right)}_{A}}{C_{IS}} \end{array}$$

Where *m*_*A*_ and *m*_*IS*_ are calibration curve sensitivities for analyte and internal standard, respectively, and *C(std)*_*A*_ is the concentration of analyte in the standard solution.

Because both *C(std)*_*A*_ and *C*_*IS*_ are known and constant (as they were added to S1), *C(sam)*_*A*_ is readily calculated from the SDA calibration curve equation using Equation (6). Considering that *C(std)*_*A*_ = *C*_*IS*_ = 50 μg/L in the example shown in Figures 4 and 5, the concentrations of Ba and Cu in solution are 8.22 and 71.5 μg/L, respectively. These values correspond to 8.22 and 71.5 mg/kg in the sample replicate analyzed (0.2499 g of sample digested, with solution volume made up to 25.0 mL and then diluted 10-fold before analysis), and recoveries of 95.6 and 99.9%, respectively.

#### Multi-isotope calibration (MICal) and multispecies calibration (MSC)

Both MICal and MSC are based on a dimension rarely explored in calibration, which is related to the capability of modern instruments to simultaneously, or at least fast-sequentially, monitor several analytical signals. Different from a traditional calibration method, which is based on determining a mathematical function relating analyte concentration and instrument response at a specific region of the spectrum, MICal and MSC use the instrument response from a single analyte concentration recorded at multiple points of the spectrum for calibration. Although applied for ICP-MS analysis, both MICal and MSC are an extension of the multi-energy calibration method (MEC), which has been recently applied in ICP OES, MIP OES and HR-CS F AAS (Virgilio et al., 2017). As with SDA, MEC, MICal and MSC rely on a matrix-matching strategy to minimize matrix effects. Calibration solutions are prepared in the same fashion as S1 and S2 in SDA, except they are run separately in MEC, MICal and MSC and no internal standard is required. The calibration plot is built with signals recorded for S1 and S2 on the *x*-axis and *y*-axis, respectively. Each point in the calibration plot corresponds to a different wavelength (MEC; Virgilio et al., 2017), isotope (MICal; Virgilio et al., 2018), or molecular ion (MSC; Williams and Donati, 2018) of the same analyte; all produced from a single analyte concentration, which was added to S1.

Because MEC, MICal and MSC rely on the same principle, they can be mathematically described using the same equations. Consider the following functional relationships for S1 and S2 (Equations 7 and 8, respectively), where *S(X*_*i*_*)*_*Sam*+*Std*_ and *S(X*_*i*_*)*_*Sam*_ are the instrument responses for a given (*X*_*i*_) analytical signal source (i.e., wavelength, isotope or molecular ion); *m* is a proportionality constant; and *C(A)*_*Sam*_ and *C(A)*_*Std*_ are the analyte concentrations in the sample and in the standard solution added to S1.

(7) $$\begin{array}{r} S{\left(X_{i}\right)}_{Sam+Std}=m\;\left[C{\left(A\right)}_{Sam}+C{\left(A\right)}_{Std}\right] \end{array}$$

(8) $$\begin{array}{r} S{\left(X_{i}\right)}_{Sam}=mC{\left(A\right)}_{Sam} \end{array}$$

Given the same set of instrumental conditions and the same matrix composition since both S1 and S2 contain the same amount of sample, *m* will have the same value in both Equations (7) and (8), which may then be combined (Equation 9), and rearranged to give Equation (10).

(9) $$\begin{array}{r} \frac{S{\left(X_{i}\right)}_{Sam}}{C{\left(A\right)}_{Sam}}=\;\frac{S{\left(X_{i}\right)}_{Sam+Std}}{C{\left(A\right)}_{Sam}+C{\left(A\right)}_{Std}} \end{array}$$

(10) $$\begin{array}{r} S{\left(X_{i}\right)}_{Sam}=S{\left(X_{i}\right)}_{Sam+Std}\left[\frac{C{\left(A\right)}_{Sam}}{C{\left(A\right)}_{Sam}+C{\left(A\right)}_{Std}}\right] \end{array}$$

By plotting *S(X*_*i*_*)*_*Sam*+*Std*_ (from S1) on the *x*-axis, and *S(X*_*i*_*)*_*Sam*_ (from S2) on the *y*-axis, with multiple signal sources of the same analyte (*X*_*i*_*, X*_*j*_*, X*_*k*_, …, *X*_*n*_) corresponding to different points on the calibration graph, the slope of the linear regression model will be:

(11) $$\begin{array}{r} Slope=\;\left[\frac{C{\left(A\right)}_{Sam}}{C{\left(A\right)}_{Sam}+C{\left(A\right)}_{Std}}\right] \end{array}$$

and because *C(A)*_*Std*_ is known, the analyte concentration in the sample may be easily determined by rearranging Equation (11), which leads to Equation (12).

(12) $$\begin{array}{r} C{\left(A\right)}_{Sam}=\frac{Slope\cdot C{\left(A\right)}_{Std}}{\left(1-Slope\right)} \end{array}$$

**Figure 6:**
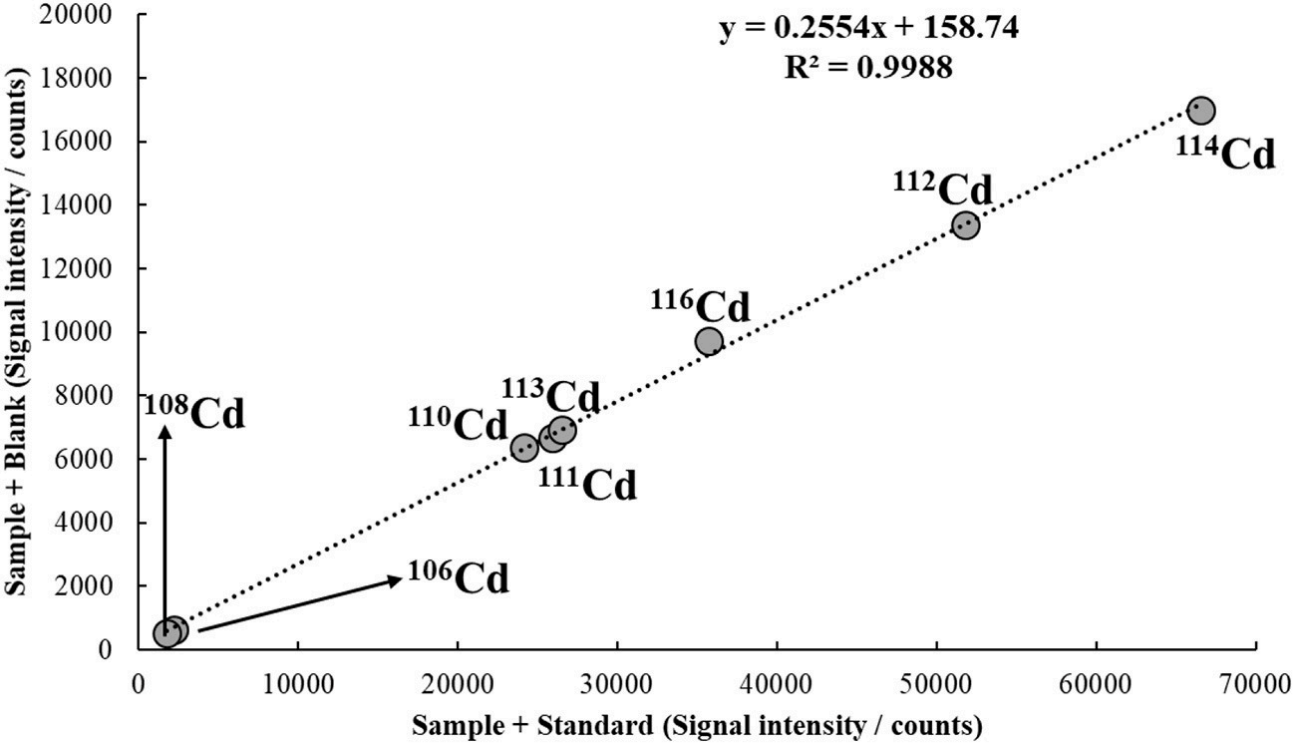
MICal plot used to determine Cd in a tap water sample spiked with 10 μg/L of the analyte. The concentration of Cd standard added to S1 [*C(A)*_*Std*_] was 30 μg/L. He flowing at 3.5 mL/min was used in the collision/reaction cell.

A typical MICal calibration plot is depicted in Figure 6. In this example, a tap water sample spiked with 10 μg/L of Cd is analyzed by ICP-MS. By applying Equation (12) and considering a [*C(A)*_*Std*_] of 30 μg/L, the concentration of Cd in the sample is calculated as 10.3 μg/L, i.e., a recovery of 103%.

For simplicity, a 1:1 mixture of sample and standard solution (S1), and sample and blank (S2) is frequently used in MEC, MICal and MSC. However, any other proportion may be used, as long as both S1 and S2 contain the same amount of sample. A 20% sample/80% standard solution (or blank) may be adopted, for example, to further minimize matrix effects; or a 70% sample/30% standard solution (or blank) may be used to improve sensitivity. In such cases, however, a dilution factor must be included in Equation (12), as shown in Equation (13), where V_Std_ corresponds to the volume of standard solution added to S1, and V_sam_ is the volume of sample added to both S1 and S2 (Virgilio et al., 2018).

(13) $$\begin{array}{r} C{\left(A\right)}_{Sam}=\frac{Slope\cdot C{\left(A\right)}_{Std}\cdot V_{Std}}{\left(1-Slope\right)\cdot V_{Sam}} \end{array}$$

**Figure 7:**
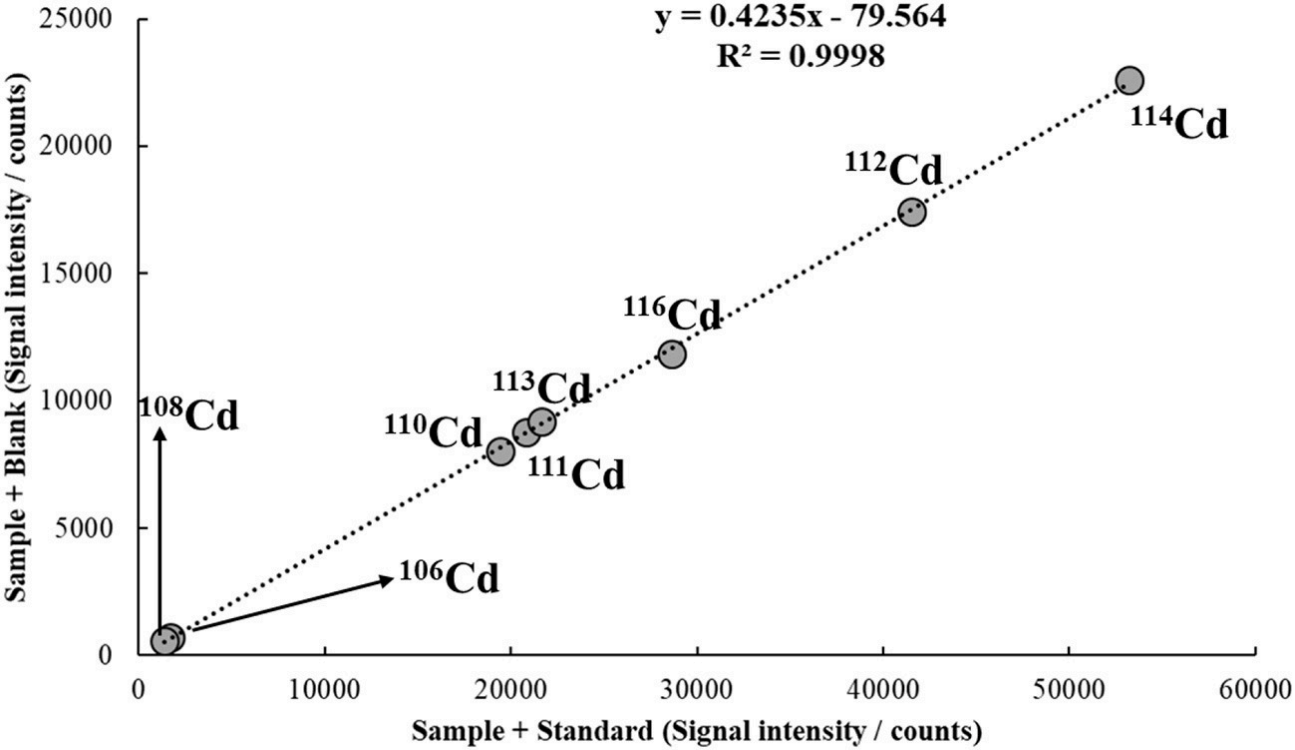
MICal plot used to determine Cd in a tap water sample spiked with 10 μg/L of the analyte. S1 and S2 were prepared with 70% of sample and 30% of standard solution or blank. The concentration of Cd standard added to S1 [*C(A)*_*Std*_] was 30 μg/L. He flowing at 3.5 mL/min was used in the collision/reaction cell.

As an example of application using Equation (13), Figure 7 shows the MICal plot to determine Cd in tap water similar to the one discussed in Figure 6. In this case, however, S1 and S2 were prepared with 70% sample and 30% of standard solution or blank. Considering the same *C(A)*_*Std*_, i.e., 30 μg/L, the concentration of Cd in solution calculated from Equation (13) is 9.44 μg/L, which corresponds to a 94% recovery from the spiked value of 10 μg/L. Note that if *V*_*Std*_ = *V*_*Sam*_, i.e., a 1:1 mixture is used in S1 and S2, Equation (13) simplifies to Equation (12).

### Applications, advantages, and potential limitations of IFS, SDA, MICal and MSC

Tables 4 and 5 list recent applications, and the advantages and potential limitations of each of the non-traditional calibration methods discussed here. Of the four strategies discussed, IFS and SDA are the more mature methods, whereas MICal and MSC have been recently published in the literature as proofs-of-concept. The IFS method is the only non-matrix-matching approach, but it is also the only one capable of minimizing spectral interferences. It requires no instrumental modification beyond simple ICP-QMS, nor the addition of an internal standard to the sample matrix. Since its original description in the literature in 2011, the IFS method has been successfully applied to the determination of several analytes in different sample matrices (Amais et al., 2011, 2012, 2014; Donati et al., 2011, 2012a,b; Amaral et al., 2013; Latorre et al., 2015; Table 4). Although the magnitude of the IFS signal has no effect on accuracy, its fluctuation during the analysis (*V*_*IFS*_) may negatively impact analyte recoveries (first part of Equation 2; Amais et al., 2014). Such effect is due to the low resolution of the ICP-QMS, i.e., the IFS signal divides the unresolved analyte plus interfering ion signal. Therefore, a large *V*_*IFS*_ value (e.g., 30%) will result in poor recoveries (e.g., 76.9%) even if *V*_*IFS*_ = V_I_, according to Equation (2). Fortunately, this is rarely the case in modern ICP-MS instruments and for the relatively high IFS signals (low RSDs) used for calibration.

**Table 4:** Applications of the non-traditional calibration methods and listed references.

**Calibration method**	**Analytes**	**Samples**	**References**
IFS	As, Fe, K, Mn, P, S, Si	SRMs 1515^a^, 1548a^b^, 1577b^c^, 1643e^d^ and 1848^e^, meat, grains, diesel^f^, biodiesel, wines, and plants	Amais et al., 2011, 2012, 2014; Donati et al., 2011, 2012a,b; Amaral et al., 2013; Latorre et al., 2015
SDA	Al, As, Cd, Co, Cr, Cu, Fe, Ni, Pb, Se, Zn	Beverages, foodstuffs, food dye, concentrated acids, and mouthwash	Jones et al., 2015; Fortunato et al., 2016, 2017; Gonçalves et al., 2016b; Virgilio et al., 2016a,b; Althoff et al., 2017
MICal	Ba, Cd, Se, Sn, Zn	CRMs 1515^a^, 1547^g^, 1567a^h^, 1568b^i^, 1570a^j^, 1573a^k^, and 1643e^d^	Virgilio et al., 2018
MSC	As, Co, Mn	CRM 1573a^k^ and 1577b^c^, rice, bovine liver, and pork liver	Williams and Donati, 2018

a Apple leaves.

b Typical diet.

c Bovine liver.

d Trace elements in water.

e Lubricating oil.

f Reference sample from the Center of Characterization and Development of Materials (CCDM).

g Peach leaves.

h Wheat flour.

i Rice flour.

j Spinach leaves.

k *Tomato leaves*.

**Table 5:** Advantages and potential limitations of IFS, SDA, MICal and MSC, and the instrumental methods in which they have been used.

**Calibration method**	**Advantages**	**Potential limitations**	**Instrumental method**
IFS	Accounts for variations in interfering signals and contributes to significantly minimizing spectral interferences	IFS and interfering species must behave similarly; it cannot resolve matrix effects; there is no universal IFS species, and method development is required to identify the optimal species	ICP-MS
SDA	Combines SA and IS. It is effective at minimizing matrix effects and fluctuations due to changes in sample size, orientation, and instrumental parameters. Only two solutions are required for calibration	Only a few elements may be determined for sequential methods such as ICP-MS; automatically defining the SDA region used for calibration is not straightforward	UV-Vis, FAAS, MIP OES, ICP OES, ICP-MS, and Raman spectroscopy
MICal	Matrix-matching approach requiring less reagents and solution preparation than SA. Spectrally interfered isotopes are easily identified. Only two solutions are required for calibration	Monoisotopic elements cannot be determined; systematic errors in solution preparation are more critical than in EC, IS and SA	ICP-MS
MSC	Matrix-matching approach requiring less reagents and solution preparation than SA. Spectrally interfered species are easily identified. Only two solutions are required for calibration	Requires more complex and expensive instrumentation (ICP-MS/MS); systematic errors in solution preparation are more critical than in EC, IS and SA	ICP-MS/MS

On the other hand, the efficiency of the IFS method is directly related to how well *V*_*IFS*_ matches the signal fluctuation of the interfering species (*V*_*I*_) (Donati et al., 2011; Amais et al., 2014). If *V*_*IFS*_ is similar to *V*_*I*_, accurate results will be achieved even if the interfering signal is significantly higher than the analytical signal (note that there is no requirement for the intensities of interfering and IFS species, *S*_*I*_ and *S*_*IFS*_, to match according to Equation 2). For example, if *V*_*I*_ and *V*_*IFS*_ are 5 and 4.9%, respectively, and *S*_*I*_/*S*_*A*_ = 10, the analyte recovery will be 96.3% (Equation 2). Using the same example, if *V*_*I*_ = 5% and *V*_*IFS*_ = 1%, the analyte recovery will be 138.6%. Alternatively, if *V*_*I*_ = *V*_*IFS*_ = 5%, the analyte recovery will be 95.2% even if *S*_*I*_/*S*_*A*_ = 10^6^. Therefore, in order to obtain optimal results using the IFS method, (*V*_*I*_–*V*_*IFS*_) must be small if *I*_*I*_*/I*_*A*_ is large; or *I*_*I*_*/I*_*A*_must be small if (*V*_*I*_–*V*_*IFS*_) is large. However, potential consequences of these limitations are easily identified by the analyst, who can choose the most appropriate IFS species based on results from method development experiments. The entire procedure is usually easy since both analytical and IFS signals are monitored during analysis and one can decide whether to use the analyte/IFS signal ratio for calibration after the run.

SDA, MICal and MSC are calibration strategies used to minimize matrix effects rather than to correct for spectral interferences. Therefore, different from IFS, an additional procedure to eliminate spectral interferences is commonly required when employing these methods (e.g., He has been used as collision gas in the examples shown in Figures 4–7). SDA, MICal and MSC are relatively easy to implement, and require less reagents and allow for higher sample throughputs when compared to the traditional SA method. Because the same amount of sample is present in the calibration solutions, matrix effects are not of concern. Thus, SDA, MICal and MSC accuracies are comparable and often better than the traditional EC, IS, and SA methods (Jones et al., 2015; Virgilio et al., 2018; Williams and Donati, 2018). SDA may be more broadly applicable than MICal and MSC, as it has been successfully used in UV-vis, FAAS, MIP OES, ICP OES, and Raman spectroscopy in addition to ICP-MS (Jones et al., 2015; Fortunato et al., 2016, 2017; Gonçalves et al., 2016b; Virgilio et al., 2016a,b; Althoff et al., 2017; Table 4). Because it employs an internal standard, it is also capable of correcting for signal fluctuations due to instrument variations. However, its gradient dilution nature is a limiting factor in applications involving sequentially collected data such as in ICP-MS and MIP OES (Gonçalves et al., 2016b; Virgilio et al., 2016a; Althoff et al., 2017). With a fast dilution process (when S1 and S2 are mixed in the same container), the larger the number of analytes the fewer the points available for calibration in the negative slope region of the SDA plot (Figure 4). Therefore, different from a simultaneous SDA-ICP OES measurement, in which many calibration points are available, ICP-MS determinations are limited to a few SDA points (Figure 5). This limitation may be overcome by using an ICP-(TOF)MS, for example. Another minor difficulty encountered in SDA applications is associated with data processing. Establishing a fast, systematic procedure to automatically define the SDA region and use its data points for calibration is less than straightforward. However, computer algorithms and hardware automation may be easily implemented, which can significantly streamline the entire SDA process. An instrument setup including, for example, an automatic sampler with seamless solution mixing, and data processing software to select the SDA region and automatically calculate the analyte concentration in the sample may contribute to a more broad adoption of SDA. This is a critical need to allow for SDA and other strategies discussed here to be effectively used in routine analysis.

A significant amount of effort has been placed toward resolving spectral interferences in ICP-QMS determinations, with collision/reaction cells (CRCs) and ICP-MS/MS representing the current state of the art (Tanner et al., 2002; Koppenaal et al., 2004; Bolea-Fernandez et al., 2015, 2017; Lum and Sze-Yin Leung, 2016). Despite these efforts, ICP-MS is still susceptible to relatively intense matrix effects, which may be caused by space charge effects, or stem from samples containing organic solvents or high concentrations of acids, carbon species, and easily ionizable elements (Gajek and Choe, 2015; García-Poyo et al., 2015; Leclercq et al., 2015). Although SDA and the traditional SA method are both efficient at minimizing matrix effects, the former is limited by the sequentially recorded ICP-MS data and the restrictions on the number of analytes determined in each run (Virgilio et al., 2016a), and the latter is time and reagent consuming. On the other hand, MICal and MSC have been specifically developed for ICP-MS applications (Table 4). They are more easily implemented than SDA, as no fast dilution process is required, and common automatic sampler devices can be used for calibration. An additional advantage of using these methods when compared with SDA and the traditional EC, IS, and SA is that spectral interferences may be easily identified as an outlier point on the linear region of the calibration curve (Virgilio et al., 2017, 2018; Williams and Donati, 2018). On the other hand, systematic errors during solution preparation are not as easily detected as in the traditional methods. Thus, additional caution must be taken when preparing the standard solution added to S1 to prevent any systematic bias. Some other limitations include MICal's requirement for analytes presenting at least two stable and sensitive isotopes, and MSC's application only to instruments with MS/MS capabilities (Virgilio et al., 2018; Williams and Donati, 2018). Therefore, MICal is not applicable to monoisotopic analytes such as As, Mn and P. In a sense, MSC complements MICal, as molecular ions of monoisotopic analytes formed in the CRC of an ICP-MS/MS instrument are used for calibration. In the recently published proof-of-concept work by Williams and Donati (2018), signals from oxide and ammonia cluster ions of As, Co and Mn are used as calibration points in the MSC graph. However, in principle, any ion containing the analyte can be used in MSC. The method was successfully used to analyze CRMs of tomato leaves and bovine liver, with accuracies comparable to and often better than EC, IS, and SA. Commercial samples of rice and liver were also analyzed to demonstrate the applicability of the method in routine analyses.

### Boundaries of application for MICal and MSC

SDA is similar to the traditional calibration methods regarding its application boundaries, i.e., it may be effectively used within the linear dynamic range of a given analyte at a certain wavelength (or for a certain isotope). On the other hand, neither MICal nor MSC follow this precept, as both are based on analytical signals from multiple sources and with different sensitivities. By comparing Figures 6 and 7, it can be seen that the higher the analyte concentration in the sample [*C(A)*_*Sam*_] the larger the MICal (or MSC) slope. From Equation (11), one can observe that as *C(A)*_*Sam*_ becomes significantly higher than the analyte concentration in the standard added to S1 [*C(A)*_*Std*_], the slope approaches a value of 1. In this case, *C(A)*_*Std*_ becomes negligible as compared with *C(A)*_*Sam*_, which can compromise the accuracy of the method (Virgilio et al., 2018; Williams and Donati, 2018). Alternatively, if *C(A)*_*Sam*_ is significantly lower than *C(A)*_*Std*_, accuracy will also be compromised, as the slope will approach zero. Therefore, both MICal and MSC will perform at their best when the calibration curve slopes are away from extreme values (i.e., away from 0 and 1; Virgilio et al., 2018; Williams and Donati, 2018).

One can establish the application boundaries (or working ranges) for MICal and MSC based on expected or ideal calibration curve slopes. It is possible then to determine the minimum *C(A)*_*Std*_ and *C(A)*_*Sam*_ values that will ensure the desired slope range. Consider, for example, a working range with slopes between 0.1 and 0.9. By plugging these values into Equation (12), the lower and higher boundaries will correspond to the relationships shown in Equations (14) and (15), respectively.

(14) $$\begin{array}{r} C{\left(A\right)}_{Sam}=0.111C{\left(A\right)}_{Std} \end{array}$$

(15) $$\begin{array}{r} C{\left(A\right)}_{Sam}=9C{\left(A\right)}_{Std} \end{array}$$

An additional restriction in applying the MICal and MSC methods relates to the limits of quantification (LOQ). The minimum value for *C(A)*_*Sam*_ must be ≥ LOQ. Thus, replacing *C(A)*_*Sam*_ with LOQ in Equation (14) gives Equation (16):

(16) $$\begin{array}{r} C{\left(A\right)}_{Std}=9\text{LOQ} \end{array}$$

Therefore, *C(A)*_*Std*_ ≥ 9 LOQ and *C(A)*_*Sam*_ ≥ LOQ will ensure a minimum slope of 0.1. The higher application boundary can then be defined by combining Equations (15) and (16): *C(A)*_*Sam*_ = 81 LOQ. Thus, if *C(A)*_*Std*_ = 9 LOQ, analyte concentrations in the sample between 1 LOQ and 81 LOQ can be effectively determined, as 0.1 ≤ slope ≤ 0.9. Experimentally, the sample can be diluted, or the standard solution concentration can be adjusted to ensure these values. A practical strategy for doing that is to compare the analytical signal intensities from a given source (i.e., isotope or complex ion containing the analyte) for sample and standard solution. Considering that analytical signal intensity [*S(A)*] and analyte concentration [*C(A)*] are proportional, Equations (14) and (15) can be rewritten as *S(A)*_*Sam*_/*S(A)*_*Std*_ = 0.111, and *S(A)*_*Sam*_/*S(A)*_*Std*_ = 9. Thus, one can run the sample and the standard solution separately and compare their analytical signal intensities for the most sensitive isotope or the most sensitive analyte ion complex. If their signal ratio is between 0.111 and 9, the calibration curve slope should be approximately in the 0.1–0.9 range. It is important to note that this measurement is only approximate because sensitivity differences are expected between sample and standard solution due to matrix effects. However, it allows for a fast semi-quantitative assessment of the analyte concentration in the sample and contributes to the decision of whether concentration adjustments are necessary.

## Conclusions

The ideal calibration method is cost-effective, simple, widely applicable, and capable of correcting for spectral and non-spectral interferences, which results in significant improvements in precision, accuracy and sample throughput. However, as in many other contexts, there is no such thing as a *one-size-fits-all* calibration method. Different strategies, or a combination of methods may be used with complex-matrix samples, and instrumental or sample preparation strategies may be required when severe interfering effects are present.

**Figure 8:**
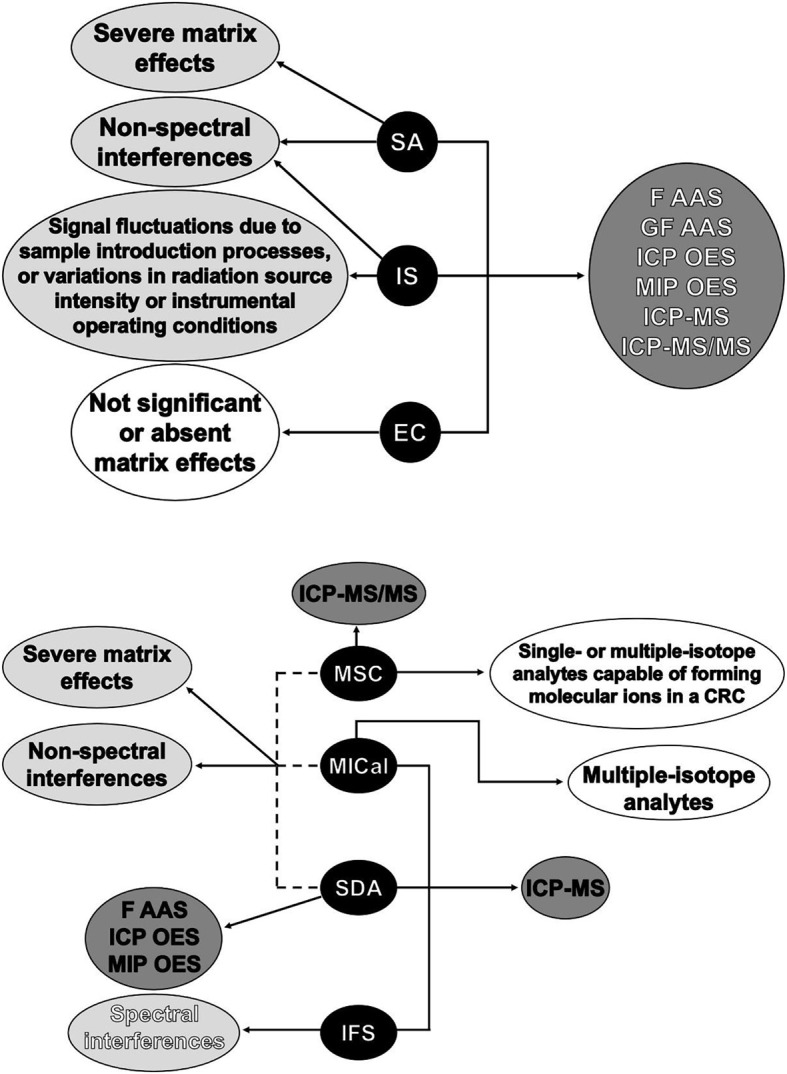
Summary of the most adequate general applications for EC, IS, SA, IFS, SDA, MICal, and MSC.

We hope this review paper has demonstrated that calibration is essential to any analytical procedure, and the analyst always needs to consider its effects on accuracy, precision and sample throughput when determining the most appropriate method for a given application. As discussed here, a carefully chosen calibration method can improve the analytical performance of an instrumental spectrochemical technique. It may be a simple, fast and cost-effective alternative to instrumental solutions dealing with spectral and non-spectral interferences. A summary of the most adequate general applications for each of the calibration methods discussed in this work is shown in Figure 8. As analytical instrumentation evolves, new calibration approaches based on innovative data processing strategies should be developed to support faster and more accurate procedures, which will ultimately contribute to a better understanding of the role of trace elements in chemical processes, human health and the environment.

## Author contributions

JC and AB performed the general literature review. AB and JN prepared the traditional calibration methods section. JC and GD prepared the non-traditional calibration methods section. JN and GD edited the manuscript.

### Conflict of interest statement

The authors declare that the research was conducted in the absence of any commercial or financial relationships that could be construed as a potential conflict of interest.
